# Enhancing virtual screening of cystathionine β-synthase inhibitors: benchmarking target-specific machine-learning scoring functions against state-of-the-art AI docking and co-folding approaches

**DOI:** 10.1186/s13321-026-01262-x

**Published:** 2026-07-18

**Authors:** Cao-Minh Truong, Van-Thinh To, Nathalie Janel, Julien Dairou, Pedro J. Ballester, Olivier Taboureau, Viet-Khoa Tran-Nguyen

**Affiliations:** 1 Université Paris Cité, CNRS UMR 8251, INSERM ERL 1133, 75013 Paris, France; 2 Université Paris Cité, CNRS UMR 8251, 75013 Paris, France; 3 Université Paris Cité, CNRS UMR 8601, 75006 Paris, France; 4 Department of Bioengineering, Imperial College London, London, SW7 2AZ UK

**Keywords:** Cystathionine β-synthase, Inhibitor, Machine learning, Target-specific, Binary classification, Deep-learning docking, Co-folding, Benchmark, Virtual screening

## Abstract

Cystathionine β-synthase (CBS) has emerged as an important therapeutic target implicated in cancer and Down syndrome, yet the discovery of selective CBS inhibitors remains challenging due to limited structural diversity of known ligands and the scarcity of target-focused virtual screening (VS) benchmarks. In this study, we present the first comprehensive evaluation of CBS-specific artificial intelligence (AI) models for structure-based VS, supported by a carefully curated and up-to-date data set of experimentally validated CBS inhibitors, true inactives and decoys. Using this data set, we developed CBS-specific binary classification models trained on docking-derived features and evaluated them in a rigorous five-fold cross-validation framework that employed similarity-controlled splits, ensuring structural independence between training and test sets while minimizing intra-fold class bias. Predictive performance was assessed primarily using the normalized enrichment factor of true actives at 1% (NEF1%) alongside balanced accuracy. Across all folds, the CBS-specific AI models demonstrated consistently high screening power and robust classification performance. Importantly, we benchmarked their performance against a diverse panel of 16 established VS pipelines, including widely adopted docking-based scoring schemes, modern deep-learning (DL) docking tools, and recent co-folding approaches for protein–ligand binding prediction. The CBS-specific AI classifiers substantially outperformed these state-of-the-art (SOTA) methods in early enrichment (NEF1% = 0.764 ± 0.191), highlighting the advantage of target-focused training for VS tasks. Our results further reveal that generic DL docking and co-folding approaches struggle to achieve reliable screening performance when applied to targets under-represented in or entirely absent from their training data, as appears to be the case for CBS. This finding underscores a key limitation of broadly trained foundation-style models in prospective drug discovery campaigns involving less-studied proteins. Overall, this work reinforces the benefits of target-specific AI scoring models tailored to individual proteins, highlights the value of high-quality curated data sets for such efforts, and provides practical guidance for selecting VS strategies in case of data-limited targets. To promote transparency and reproducibility, all input and output files, curated data sets and source code are freely available via GitHub and Zenodo.

**Supplementary Information:**

The online version contains supplementary material available at https://doi.org/10.1186/s13321-026-01262-x.

## Scientific Contribution

We present the most comprehensive and up-to-date data set for CBS inhibitors, integrating experimental data from PubChem, ChEMBL, the scientific literature and chemical patents, which substantially enriches the active compound space for this underexplored target. For the first time, we developed CBS-specific AI binary classification models and benchmarked them against 16 existing structure-based VS pipelines, including SOTA end-to-end DL-based docking tools and co-folding approaches, demonstrating that our models consistently achieved superior early hit retrieval. Our findings also reveal the limitations of generic SOTA tools when applied to targets under-represented in or absent from their training data, highlighting the value of target-specific AI models for data-limited protein targets.

**Supplementary Information:**

The online version contains supplementary material available at https://doi.org/10.1186/s13321-026-01262-x.

## Introduction

Down syndrome (trisomy 21) is a congenital chromosomal disorder caused by an extra copy of human chromosome 21 and is the most frequent genetic abnormality in humans, with an incidence of one in 1000 births [1]. It is characterized by intellectual disability, psychomotor delay and hypotonia, with marked interindividual variability. Genotype–phenotype correlation studies have identified critical regions on chromosome 21 whose duplication contributes to these features, including cognitive impairment. Among the therapeutic targets in these regions, cystathionine β-synthase (CBS), a key enzyme of homocysteine metabolism encoded on chromosome 21, has emerged as a candidate involved in the neurological manifestations of Down syndrome. CBS functions at the intersection of one-carbon metabolism and the transsulfuration pathway, catalyzing homocysteine and serine condensation to cystathionine [2], and is a major source of endogenous hydrogen sulfide (H₂S) via alternative reactions, including homocysteine and cysteine condensation [3, 4]. A pyridoxal-5′-phosphate (PLP)-dependent enzyme regulated by S-adenosylmethionine [5], CBS is predominantly expressed in peripheral tissues but is also present in the central nervous system, with developmentally regulated expression and high levels in the hippocampus and cerebellum [6–8]. Elevated CBS protein and activity in fibroblasts and brain tissue from individuals with trisomy 21 support a gene dosage effect [8, 9], implicating CBS dysregulation in Down syndrome pathophysiology, potentially via excessive H₂S, a gaseous neuromodulator that modulates N-methyl-D-aspartate receptor-mediated neurotransmission at physiological concentrations [10–13]. Partial pharmacological inhibition of CBS has been proposed to restore metabolic balance while avoiding the adverse effects of complete enzyme inhibition [14]. In this context, aminooxyacetic acid (AOAA), a commonly used CBS inhibitor, forms a PLP-bound oxime intermediate [15]. However, its inhibition is reversible under physiological conditions and non-selective, as it also affects numerous other enzymes, highlighting the need for more selective and controllable CBS inhibitors [15–17].

Although the CBS/H₂S pathway has gained recognition in human pathophysiology over the past decade, the development of selective and potent CBS inhibitors remains in its infancy [16]. Historically, CBS was primarily targeted for activation in homocystinuria, and pharmacological inhibition remained largely unexplored until its roles in Down syndrome and several cancers, including colorectal, ovarian and breast cancer, had emerged [17]. Moreover, CBS gene polymorphisms may influence enzyme activity and metabolic phenotypes, further supporting the potential relevance of CBS-targeted strategies in personalized medicine. In this context, the development of selective inhibitors of CBS has become an area of growing interest. AOAA remains the most commonly used inhibitor in cell-based and in vivo studies despite its limitations [16]. Several additional small molecules have been reported as CBS inhibitors, including hydroxylamine derivatives, sikokianin C, NSC67078 and CH004, as summarized in a recent review article [16]. However, many of these compounds inhibit CBS weakly, with half-maximal inhibitory concentrations (IC_50_) in the tens to hundreds of micromolar range, and some even show stronger activity against other enzymes, such as cystathionine γ-lyase (CSE), e.g., hydroxylamine (IC_50_ of 20–400 μM for CBS versus 5 μM for CSE), or myricetin (IC_50_ of 18.8 μM for CBS versus 14.4 μM for CSE) [16]. Public repositories including PubChem and ChEMBL report only a few true CBS inhibitors with IC_50_ ≤ 10 μM [18, 19], commonly regarded as highly potent [14]. This scarcity of validated inhibitors, as well as documented inactives, underscores the need to integrate data from publications and patents. Such integration is essential for developing prediction tools based on artificial intelligence (AI), i.e., computational systems capable of performing tasks requiring human-like intelligence, including machine learning (ML), a subset of AI that learns from data for predictive or decision-making tasks, and deep learning (DL), a specialized subset of ML based on multi-layered neural networks [20]. These tools rely on both true actives and inactives for robust training and evaluation. Moreover, to date, no CBS-specific AI models or scoring functions have been developed for inhibitor detection. In contrast, well-studied targets such as hERG, PD-1/PD-L1, or PARP1 have benefited from AI methodologies, which substantially improved the prediction of active compounds [21–26], highlighting a significant opportunity for innovation in the CBS field.

In light of the limited availability of potent and selective CBS inhibitors, this study aimed to construct a comprehensive, up-to-date data set of CBS actives and inactives by integrating compounds from PubChem, ChEMBL, scientific publications and chemical patents. Published studies and patents were particularly important to enrich the true active population, which was defined using a 10 μM IC_50_ threshold [14], as relying solely on PubChem and ChEMBL would have yielded too few actives. On the other hand, to address the scarcity of true non-inhibitors, we incorporated “dark chemical matter” (DCM), corresponding to recurrent inactives identified in high-throughput screening assays across multiple targets [27], as decoys (assumed inactives). This practice follows a recent strategy showing that AI models trained with such compounds closely replicate performance obtained with experimentally confirmed non-binders, providing a practical solution for low-data targets [27]. Using this data set, we implemented a five-fold cross-validation (CV) strategy in which fold composition was determined based on clustering with Uniform Manifold Approximation and Projection (UMAP), a robust splitting method for molecular data sets that improves upon traditional approaches such as Butina clustering or scaffold-based splits [28]. Leveraging physics-based docking using Smina [29], protein–ligand extended connectivity (PLEC) fingerprint encoding [30], and a range of AI algorithms, including random forest (RF), extreme gradient boosting (XGBoost), support vector machines (SVM), and multilayer perceptron (MLP), we trained and evaluated CBS-specific binary classifiers for inhibitor prediction. To the best of our knowledge, this is the first application of AI methodologies specifically for CBS inhibitor detection, leveraging the most complete set of available CBS activity data. For benchmarking, we compared our models against state-of-the-art (SOTA) DL-based docking tools including DeepDock [31], Interformer [32], SurfDock [33], and KarmaDock [34]; co-folding approaches such as Boltz-2 [35], Boltz-2x [35], and AlphaFold 3 [36]; and baseline scoring methods such as Smina [29], RF-Score-VS v.2 [37], and GNINA 1.3 [38]. Evaluation across all five folds demonstrated the enhanced screening power of target-specific AI models for CBS, even in a low-data regime, providing the first comprehensive benchmarking case study for this enzyme and highlighting the potential of combining curated data sets, AI algorithms and established docking methods for target-specific structure-based virtual screening (VS). Computational jobs reported in this study were performed on a Linux-based system equipped with an A100 GPU. All input/output data and user-friendly source code are available in our GitHub repository and on Zenodo (cf. “Data availability”).

## Results and discussion

### Computational workflow

**Fig. 1:**
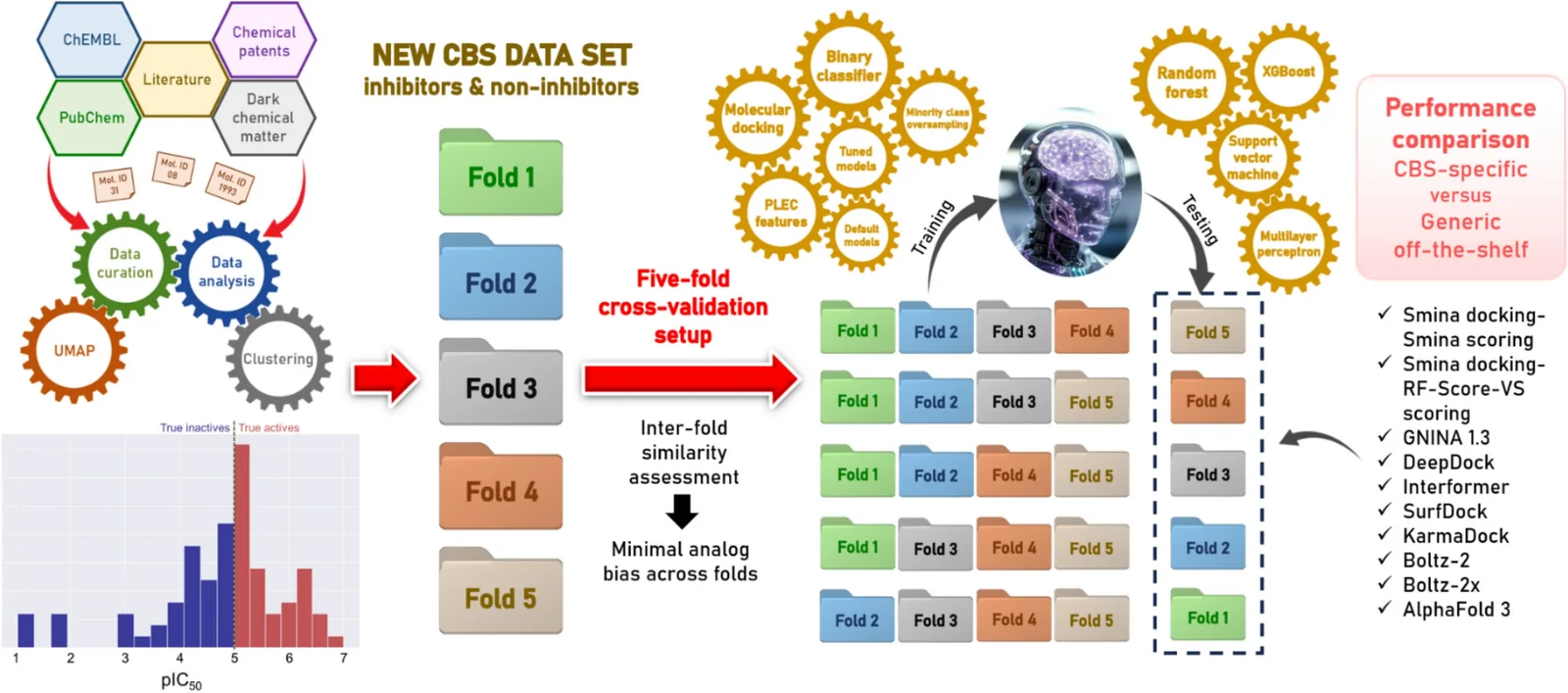
Computational workflow of our study. A series of AI-based binary classifiers for CBS inhibitor prediction were developed and evaluated using a five-fold CV setup. The models employed RF, XGBoost, SVM, and MLP as learning algorithms, with PLEC fingerprints extracted from docking poses of CBS actives and inactives curated from PubChem, ChEMBL v.36, literature, chemical patents, and the ChemDiv webserver of DCM compounds. Within this framework, multiple modeling strategies including default training, hyperparameter tuning only, minority-class oversampling only, and their combination were systematically assessed. For the tuned models, optimization was performed on the training set of each training-test split via internal four-fold CV, after which each model was retrained on the full corresponding training set using the optimal hyperparameters and decision threshold. All models were subsequently evaluated on the held-out test fold, ensuring no data leakage and minimizing inter-fold bias

The overall computational workflow of this study is illustrated in Fig. 1. Small-molecule data associated with CBS inhibitory assays including ligand identifiers (IDs), SMILES strings, and experimentally reported IC_50_ values (µM) were retrieved from PubChem and ChEMBL v.36 [18, 19]. Additional CBS inhibition data were collected from a recent review [16] and three chemical patents (WO2010072807A2, WO2010072807A3, and WO2013068592A1) [39–41]. All collected molecules underwent a rigorous curation procedure (cf. “Methods”) aimed at: (i) removing ambiguous entries (active compounds without quantitative potency values or primary assay hits lacking confirmatory measurements) and duplicate structures based on canonical SMILES; (ii) assigning a unified activity label using a stringent IC_50_ threshold of 10 µM to distinguish active from inactive compounds [14]; and (iii) eliminating potential false positives (luciferase inhibitors, promiscuous aggregators, auto-fluorescent compounds). This curation strategy follows recently published best-practice protocols [42], and is consistent with procedures adopted in LIT-PCBA [43] and our prior work [21]. To alleviate the scarcity of experimentally confirmed inactives, DCM compounds were employed as decoy molecules, in accordance with recent recommendations advocating their use as “assumed inactives” in low-inactive scenarios [27]. To minimize potential bias arising from obvious physicochemical differences between classes, only DCM compounds whose key properties simultaneously fell within the ranges observed for the true actives were retained. These properties included molecular weight (MW), atom and bond counts, lipophilicity (logP), hydrogen-bond donor and acceptor counts, rotatable bond count, and topological polar surface area (TPSA) (cf. “Methods”) [21, 43]. Care was taken to ensure no intra- or inter-class structural overlap. The true inactives and retained property-matched DCM molecules were subsequently merged and treated collectively as a single inactive class.

All compounds were then subjected to UMAP-based dimensionality reduction followed by agglomerative clustering, performed separately for actives and inactives using Morgan fingerprints (cf. “Methods”). This strategy, shown to provide more robust and less biased splits than traditional scaffold-based partitioning [28, 44], yielded structurally coherent clusters. Entire clusters were next assigned to one of five folds under predefined constraints designed to prevent data leakage, ensure overlap in physicochemical properties between active and inactive compounds within each fold, and preserve structural independence between folds (cf. “Methods”). Same-class compounds (i.e., those having the same activity label) in different folds were required to remain structurally dissimilar, enforced using a maximum pairwise Tanimoto similarity threshold of 0.70 (Morgan fingerprints, 2048 bits, radius 2), a commonly adopted cutoff for meaningful structural dissimilarity [23, 24, 42, 45–47]. This design minimized both analog bias across folds and property-based class-separation bias within folds, thereby ensuring a realistic and challenging evaluation setting. All molecules in each fold were docked into a crystallographic CBS structure obtained from the Protein Data Bank (PDB), using Smina (v.2019–10-15) [29]. Docking was performed within the experimentally defined binding site of a co-crystallized CBS inhibitor, and only one docking pose was generated per successfully docked molecule (cf. “Methods”). PLEC fingerprints were subsequently extracted from all Smina docking poses [30]. Introduced in 2019, these topological circular fingerprints are adaptations of extended-connectivity fingerprints that incorporate the atomic features of interacting protein–ligand atoms located within a specified distance cutoff [30].

Using these PLEC fingerprints as input features, we trained a series of AI binary classifiers employing four supervised learning algorithms: RF, XGBoost, SVM, and MLP, following a five-fold CV strategy. This CV design provided several advantages: (i) each fold served once as external test data, maximizing the utility of a limited data set; (ii) model robustness and generalizability were assessed across multiple independent training-test partitions instead of only one; and (iii) benchmarking against existing VS tools was rendered more rigorous and reliable, as performance was averaged over multiple structurally distinct test sets rather than using a single, small, and bias-prone split that might be subject to random variability. For each of the five training-test splits (four folds for training, the remaining fold for testing), 16 models were constructed:

(i) Default models: eight binary classifiers (RF, XGBoost, SVM, and MLP) were trained using default hyperparameters with the classification threshold fixed at 0.50. For each algorithm, two variants were constructed: one trained with oversampling of the minority class (active compounds) to match the number of inactives, and one trained without oversampling.

(ii) Tuned models: eight additional binary classifiers (RF, XGBoost, SVM, and MLP) were developed with hyperparameter optimization performed exclusively on the training data via an internal four-fold CV procedure (preserving the predefined fold composition). Hyperparameters were selected by maximizing the normalized enrichment factor of true actives in the top 1% (NEF1%), a metric directly reflecting early-stage screening performance and relevant for practical VS applications. This optimization strategy was chosen because the primary objective of the models was VS rather than conventional classification performance [48]. Following hyperparameter selection, the decision threshold (for the classification task) was also optimized using the same internal CV setup and training data, maximizing the balanced accuracy. As with the default setting, two variants per algorithm were generated: one incorporating active-class oversampling during both hyperparameter and threshold optimization, and one without oversampling. The final tuned models were retrained on the complete training set.

All 16 trained classifiers were applied to the held-out fold, used as external test data, where both classification performance and screening power were assessed. To contextualize the performance of our CBS-specific models, we conducted a comprehensive benchmarking study on each of the five folds, evaluating:

(i) Baseline VS pipelines: Smina scoring on Smina-generated poses [29], RF-Score-VS v.2 rescoring of Smina poses [37], and convolutional neural network (CNN)-Score scoring of GNINA 1.3 poses [38], representing established physics-based or hybrid AI-enhanced docking pipelines;

(ii) SOTA end-to-end DL-based docking tools: DeepDock [31], Interformer [32], SurfDock [33], and KarmaDock [34];

(iii) SOTA co-folding approaches for protein–ligand binding prediction: Boltz-2 [35], Boltz-2x [35], and AlphaFold 3 [36].

For all tools, including our AI models, screening power was quantified using the NEF1% on each fold. In addition, for our binary classifiers, balanced accuracy was reported to account for the pronounced class imbalance in the data set [49].

### Data retrieval, data curation and stratified data split

**Fig. 2:**
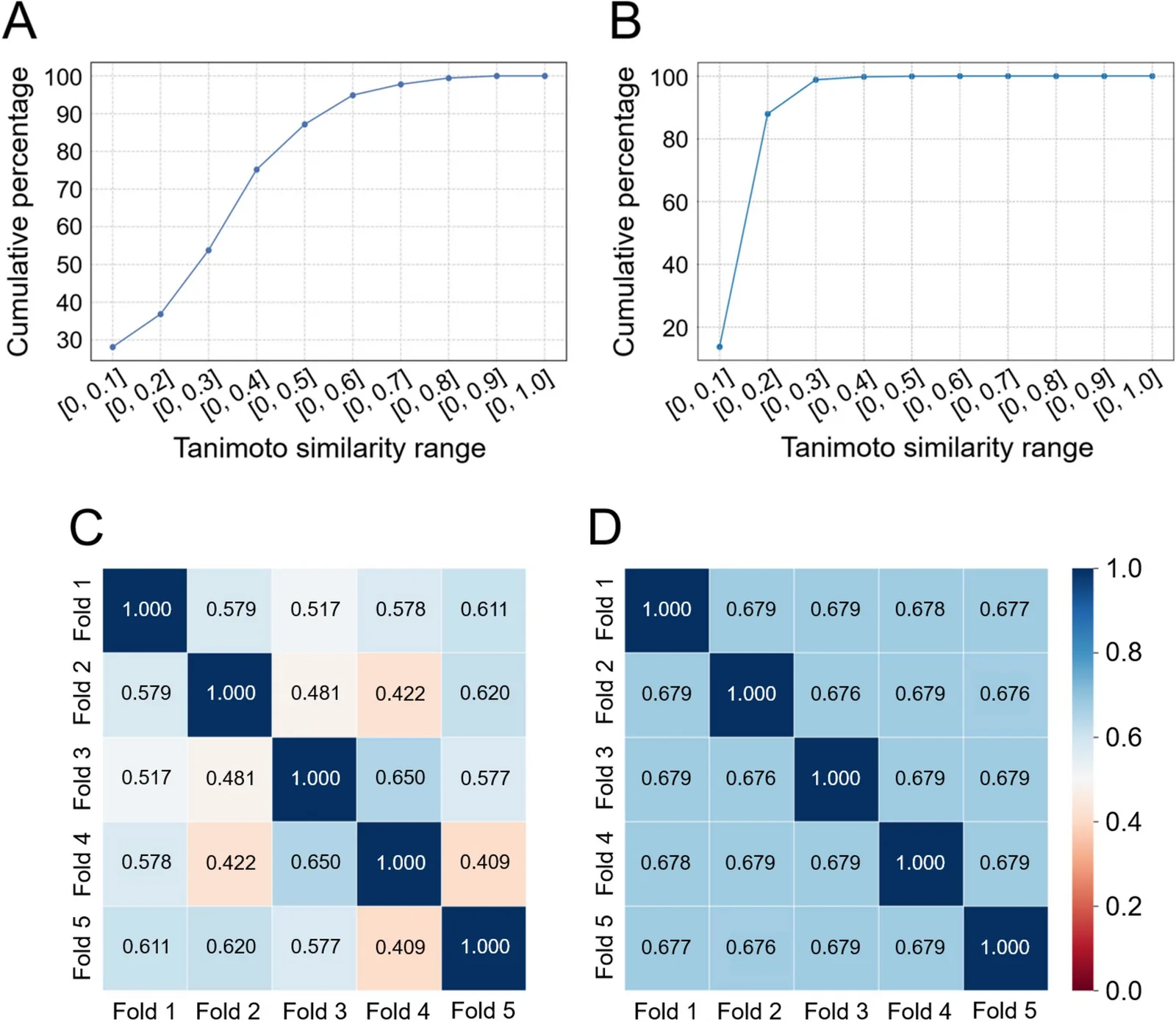
Structural diversity of our CBS-specific data set. **A, B** Cumulative percentage of active pairs (**A**) and inactive pairs (**B**) whose Tanimoto similarity fell within defined ranges, in terms of Morgan fingerprints (2048 bits, radius 2). Only 2.21% of active pairs exceeded a similarity of 0.70, while nearly 99% of inactive pairs displayed similarities ≤ 0.30, indicating high structural diversity. **C, D** Heatmaps of maximum pairwise Tanimoto similarity (Morgan fingerprints, 2048 bits, radius 2) between compounds of the same class across different folds for actives (**C**) and inactives (**D**). No same-class compounds across folds exceeded a similarity of 0.70, confirming strong structural separation between folds and supporting an unbiased and rigorous five-fold CV framework for benchmarking our CBS-specific AI models against SOTA VS tools

A total of 10,445 compounds, comprising 43 true CBS inhibitors (experimental IC_50_ ≤ 10 μM) and 10,402 inactive molecules (195 experimentally confirmed inactives and 10,207 retained DCM compounds), constituted the final data set used in this study (cf. “Methods”). Although the active set was relatively small, reflecting both the early stage of selective CBS inhibitor development and the stringent IC_50_ threshold applied to define true actives [14, 16], it nevertheless displayed substantial structural diversity (Fig. 2A). Specifically, among the 903 possible pairwise combinations of true actives, only 2.21% exhibited a pairwise Tanimoto similarity greater than 0.70 (Morgan fingerprints, 2048 bits, radius 2). The inactive population was even more diverse: among over 54 million inactive-inactive pairs, nearly 99% showed a Tanimoto similarity ≤ 0.30, compared with < 95% when only experimentally confirmed inactives were considered. Thus, the incorporation of DCM compounds as “assumed inactives,” following recent recommendations [27], helped enhance the structural diversity of our inactive pool (Fig. 2B). Moreover, the retained DCM compounds were selected to match the ranges of all eight physicochemical properties of the 43 true actives, ensuring property comparability between classes (Figure S1, Supporting Information).

The subsequent UMAP-based dimensionality reduction followed by agglomerative clustering procedure yielded 11 active clusters and 2570 inactive clusters, with silhouette scores of 0.94 and 0.97, respectively, indicating excellent cluster quality characterized by strong intra-cluster cohesion and clear inter-cluster separation (Table S1, Supporting Information). Following cluster assignment to the five folds (cf. “Methods”), the final fold composition is summarized in Table 1. These folds were well balanced in size, with a consistently low active-to-inactive ratio, reflecting the class imbalance typically observed in real-world VS campaigns [42, 43]. Importantly, the physicochemical properties of actives and inactives within each fold also showed substantial overlap across all properties (Figures S2-S7, Supporting Information). Indeed, logistic regression models trained and evaluated across the five training-test splits of our framework, using ligand-derived physicochemical descriptors computed with RDKit and normalized using ‘scikit-learn’, achieved low NEF1% values ranging from 0.000 to 0.125 (Data S1, Supporting Information). This finding suggests that, within our study design, AI models are unable to rely solely on differences in ligand-derived physicochemical properties to prioritize CBS inhibitors. Additionally, the maximum pairwise Tanimoto similarity (Morgan fingerprints, 2048 bits, radius 2) between compounds of the same class across different folds never exceeded 0.70 (Fig. 2C, D; Figures S8 and S9, Supporting Information), a commonly adopted threshold to define structurally dissimilar molecules [23, 24, 42, 45–47]. Enforcing this constraint ensured a challenging and realistic evaluation setting, as prior studies have shown that target-specific scoring functions display reduced apparent screening performance when evaluated on compounds structurally dissimilar to the training data, defined using this threshold, compared with test sets containing close training-set analogs [23, 42]. Collectively, these properties confirm that our five folds were structurally distinct, minimizing both analog bias across folds and hidden inter-class, intra-fold property differences, thereby enabling a fair and rigorous comparison between our CBS-specific AI models and existing SOTA VS tools. Full details on UMAP-based clustering results and fold composition are provided in our GitHub repository and on Zenodo (cf. “Data availability”).

**Table 1:** Composition of the five CV folds obtained using UMAP-based agglomerative clustering of the final data set, showing balanced fold sizes and consistently low active-to-inactive ratios

Fold	Number of actives	Number of inactives	Total number of compounds
Fold 1	9	2080	2089
Fold 2	10	2079	2089
Fold 3	8	2080	2088
Fold 4	8	2083	2091
Fold 5	8	2080	2088
Full data set	43	10,402	10,445

### Assessment of screening power across five folds

**Fig. 3:**
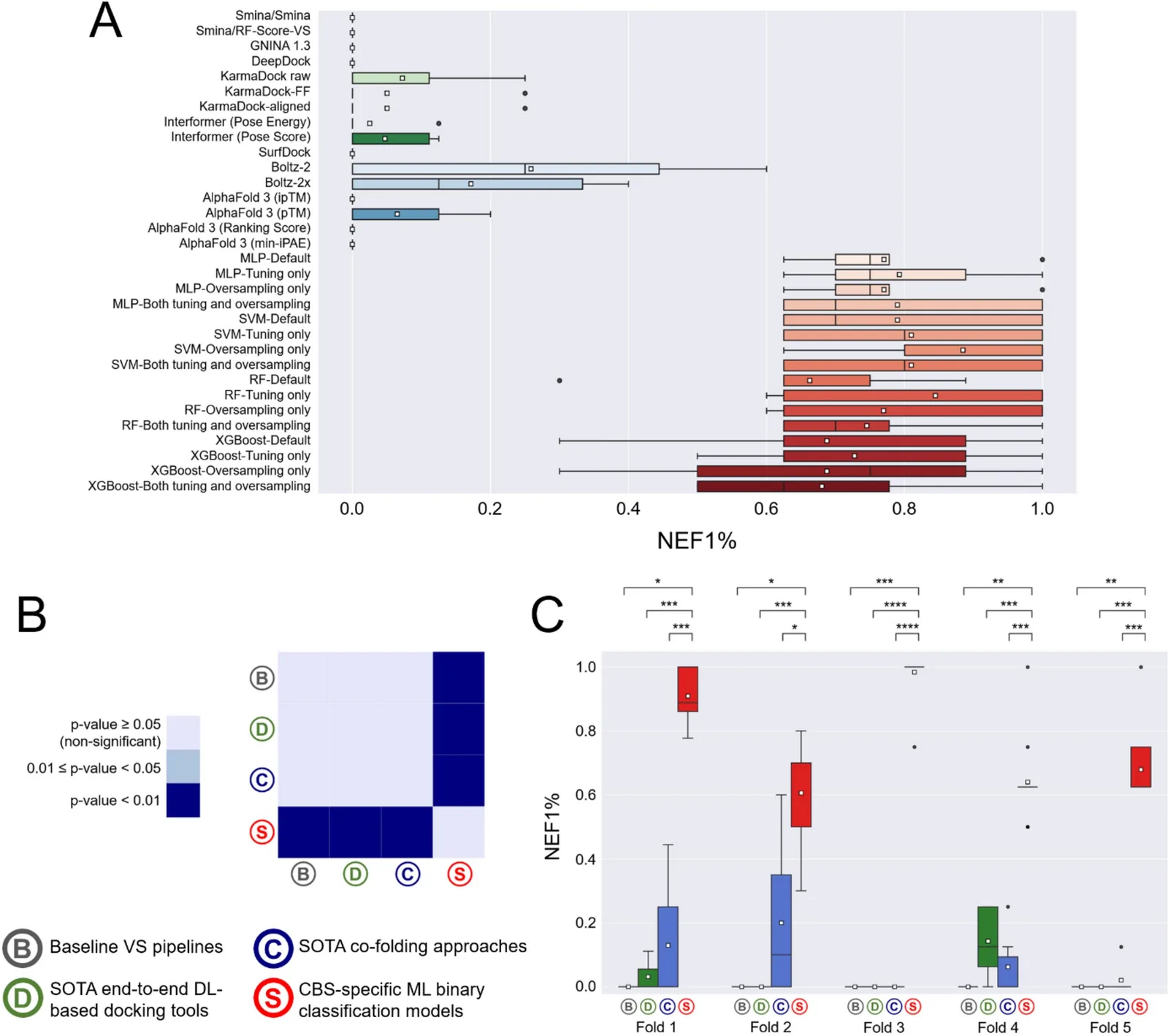
Screening power across the five folds of our CV setup for 16 existing structure-based VS pipelines and our CBS-specific AI binary classifiers based on four algorithms (RF, XGBoost, SVM, and MLP). **A** Box-and-whisker plots showing the NEF1% distribution across the five held-out test sets for all 32 evaluated VS pipelines (16 generic pipelines and 16 CBS-specific models). **B** Heatmap of pairwise p-values comparing screening power (NEF1%) between pipeline categories (baseline VS pipelines, SOTA end-to-end DL-based docking tools, SOTA co-folding approaches, and CBS-specific AI classifiers) across the five test folds. **C** Box-and-whisker plots showing per-fold screening power (NEF1%) for each VS pipeline category. For panels (**A**) and (**C**), white squares indicate mean values, the horizontal line inside each box represents the median, and filled circles denote outliers defined using the 1.5IQR rule. For panels (**B**) and (**C**), statistical comparisons were performed using the Mann–Whitney U test with Bonferroni correction, implemented via the ‘Annotator’ function from the ‘statannotations.Annotator’ module (‘statannotations’ Python package, v.0.7.2). In (**C**), only statistically significant comparisons are displayed, with significance levels indicated as follows: * (0.01 < p-value ≤ 0.05), ** (0.001 < p-value ≤ 0.01), *** (0.0001 < p-value ≤ 0.001), and **** (p-value < 0.0001). Panels (**B**) and (**C**) together show that CBS-specific AI pipelines consistently outperformed the 16 existing VS pipelines both overall and on individual folds

Screening power was evaluated using NEF1% on each fold of our five-fold CV setup for both our CBS-specific AI binary classification models and 16 existing structure-based VS pipelines (cf. “Methods,” NEF1% corresponds to EF1% normalized by its maximum attainable value on a given held-out test fold). The latter comprised baseline approaches (Smina scoring of Smina docking poses, RF-Score-VS v.2 rescoring of Smina poses, and GNINA 1.3 docking with CNN-based scoring), SOTA end-to-end DL docking tools (DeepDock; KarmaDock, with three scoring functions; Interformer, with two pose-selection scoring functions; and SurfDock), and co-folding approaches for protein–ligand binding prediction (Boltz-2; Boltz-2x; and AlphaFold 3, with four ranking metrics). Results across all five training-test splits are summarized in Fig. 3.

As shown in Fig. 3A, across all five folds of our CV design, the CBS-specific AI pipelines consistently outperformed the 16 existing VS pipelines, achieving significantly higher NEF1% values across all three categories of reference tools (Mann–Whitney U test with Bonferroni correction, p-values < 0.01; Fig. 3B). The same trend was observed when folds were examined individually, with CBS-specific models yielding superior screening performance on each held-out test set (Fig. 3C). These results are consistent with previous reports highlighting the strong screening capability of target-specific AI scoring functions in structure-based VS [21–23, 42, 50, 51]. In particular, interaction-based scoring models trained on target-relevant data, combined with physics-based pose generation, can provide more effective ligand prioritization than generic (even SOTA) tools, supporting the value of hybrid pipelines that couple conventional docking with AI-based scoring [21–23, 42]. Notably, screening (and classification) performance did not differ significantly among the four AI algorithms evaluated (RF, XGBoost, SVM, and MLP; Mann–Whitney U test, p-values > 0.05; Figure S10, Supporting Information), indicating comparable robustness across modeling approaches. Likewise, NEF1% values were statistically consistent across the five folds (p-values > 0.05; Figure S11, Supporting Information), confirming that the folds posed similar levels of difficulty. This consistency supports the effectiveness of the UMAP-based clustering and similarity-controlled splitting procedure in producing structurally independent yet comparably challenging training-test partitions, thereby enabling a fair evaluation of both our CBS-specific models and the reference VS pipelines [28]. Even when accounting for the BEDROC score and a larger top-ranked population (5%) [52], the conclusions regarding the comparative performance of our models and the existing pipelines, originally drawn from NEF1%, remain consistent (Figures S12 and S13, Supporting Information). Furthermore, when held-out-fold predictions from our 16 CBS-specific AI approaches and the 16 existing VS pipelines were concatenated, thereby enabling evaluation on the complete data as a single evaluation set, the CBS-specific approaches achieved significantly higher NEF1% than both the existing generic pipelines and the ligand-derived RDKit descriptors-based logistic regression models, in agreement with the fold-wise evaluation results (Data S1, Supporting Information). Notably, the NEF1% values of all evaluated approaches in this concatenated setting did not differ significantly from those obtained in each individual fold (Mann–Whitney U test with Bonferroni correction, p-value > 0.05). Collectively, these findings demonstrate the reliability of our study design and the consistent screening strength of CBS-specific AI pipelines for this target.

It is worth noting that the CBS-specific approaches developed in this study were trained and evaluated using a single Smina-derived docking pose per molecule. While the number of poses generated per ligand in a VS workflow remains a methodological choice, using only the top-ranked pose has been shown to yield strong screening power in similar contexts [22–24, 42], and, in the related task of binding affinity prediction, to outperform multi-pose strategies [53]. Nevertheless, it should be acknowledged that Smina does not always rank a near-native binding mode as the top-scoring solution for every active molecule, as recently demonstrated in a benchmarking study [26] (though redocking using the same protocol produced a valid pose for AOAA, the co-crystal ligand of our docking template, cf. “Methods”). Consequently, an alternative workflow could consist of sampling multiple docking poses for each ligand, followed by AI-based pose selection before CBS-specific rescoring. Such a strategy would reduce the dependence of the screening pipeline on Smina’s top-ranked pose while preserving the advantages of target-specific AI methods. Given the strong results achieved by the present pipelines, integrating an AI-based pose selection step represents a promising avenue for further improving VS performance [26].

The limited ability of the SOTA end-to-end DL docking tools and co-folding approaches to retrieve true actives within the top 1% of ranked compounds across all five held-out test sets warrants further discussion. Although these models were trained on large structural and bioactivity resources, including data sets derived from the PDB, PDBbind, PubChem, ChEMBL, and have shown good screening performance in several retrospective VS studies [54, 55], their performance on the CBS target was markedly weaker. CBS inhibitor discovery remains at an early stage [16], and experimentally characterized complexes for this protein are scarce. As of February 2026, the PDB entry 7QGT (released October 2022) represents the only human CBS holo-structure co-crystallized with an inhibitor available in the PDB [15], although other inhibitor-bound complexes from non-human organisms have also been reported (e.g., PDB IDs 3PC3, 3PC4, 6XYL). Consequently, this structure was not part of the training data used for AlphaFold 3, whose training set included those released prior to October 2021 [36]. Likewise, the training corpora reported for Boltz-2 and Boltz-2x, while extensive and incorporating data from sources such as the PDB, BindingDB, PubChem, ChEMBL together with synthetic examples [35], do not explicitly include literature- or patent-derived CBS inhibitor data. These observations suggest that such generic SOTA approaches may encounter difficulties when applied to targets that are weakly represented, or absent, in their training distributions, particularly in low-data regimes. This limitation is illustrated by Fold 3 in our study, where none of the 16 existing VS pipelines retrieved any true active within the top-ranked 1% of compounds (all NEF1% = 0.000, Table S2, Supporting Information). Notably, the actives in this fold originated exclusively from literature and patent sources, with no counterparts from ChEMBL or PubChem. Given that all folds exhibited comparable difficulty levels (Figure S11, Supporting Information), this result points to limited generalization to chemically distinct or previously unseen active series rather than to an intrinsically harder test split. Similar challenges in extrapolating to under-represented targets or ligand chemotypes have been reported for DL-based docking and co-folding structure-prediction methods in other benchmarking contexts [56–62]. Our results extend these findings to the VS setting, highlighting that strong performance observed on broadly distributed training data does not necessarily translate into reliable prioritization for underexplored targets such as CBS.

### Impact of modeling choices on screening and classification performance of CBS-specific models

**Fig. 4:**
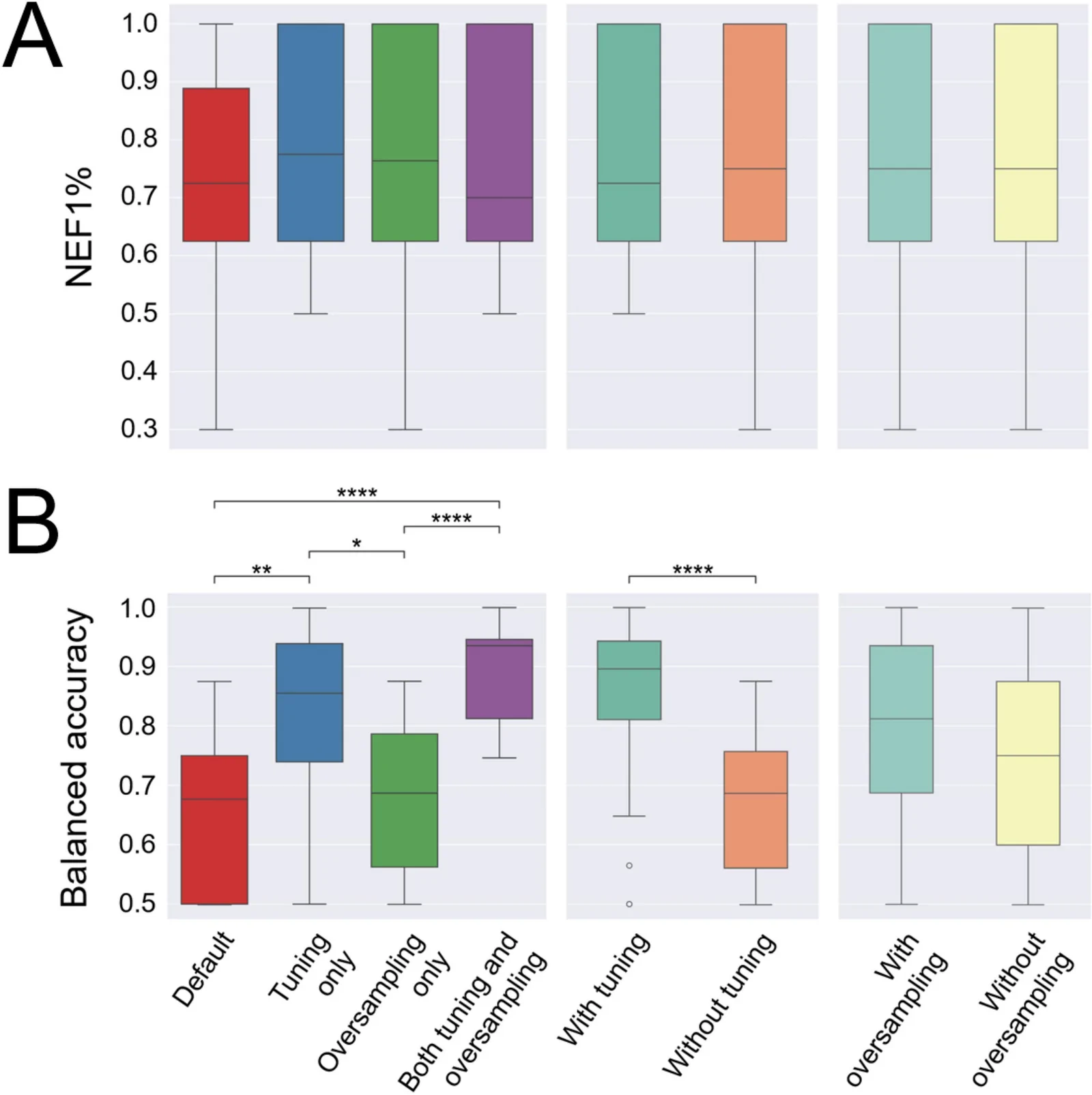
Comparison of modeling choices in terms of screening power (NEF1%, **A**) and classification power (balanced accuracy, **B**) for CBS-specific classification models across our five-fold CV setup. Statistical comparisons were performed using the Mann–Whitney U test with Bonferroni correction, implemented via the ‘Annotator’ function from the ‘statannotations.Annotator’ module (‘statannotations’ Python package, v.0.7.2). Only statistically significant comparisons are displayed, with significance levels indicated as follows: * (0.01 < p-value ≤ 0.05), ** (0.001 < p-value ≤ 0.01), *** (0.0001 < p-value ≤ 0.001), and **** (p-value < 0.0001)

We further investigated how our modeling choices influenced the screening and classification performance of the CBS-specific models across our five-fold CV setup. To this end, NEF1% and balanced accuracy values for models within the same category were grouped for comparison (Fig. 4). Specifically, we first examined the combined effect of minority-class oversampling and model tuning, considering four configurations: default models, tuning only, oversampling only, and both tuning and oversampling (left panels in Fig. 4A, B). We then evaluated each factor independently, comparing models with versus without tuning (center panels in Fig. 4A, B) and with versus without oversampling (right panels in Fig. 4A, B). Raw data for Fig. 4 are available in our GitHub repository and on Zenodo (cf. “Data availability”).

In terms of screening power (Fig. 4A), neither minority-class oversampling nor hyperparameter tuning significantly improved model performance compared with the default configurations. Although the optimization procedure used the average NEF1% from four-fold CV on the training data (for each of the five training-test splits) as the primary objective metric, the resulting screening performance did not differ significantly across models (all p-values > 0.05), including the default ones. This indicates that target-specific models trained with hyperparameters optimized for early retrieval of true actives, or trained on artificially balanced data sets, did not achieve substantially higher screening performance than those trained on the original imbalanced data without tuning. The absence of screening-power improvement from class-balanced training data is consistent with previous reports, including the recent study by Wellnitz et al. [48]. This observation is reasonable, as artificially balancing the training set leads to an active/inactive distribution significantly different from that expected in practical VS scenarios, where actives are far rarer than inactives; consequently, the original imbalanced data set better reflects real screening conditions [42, 43, 48]. Interestingly, even hyperparameter tuning explicitly aimed at optimizing early hit retrieval did not significantly improve the models’ ability to prioritize true actives in the held-out test set. It can, therefore, be inferred that selecting a CBS-specific model solely on the basis of superior retrospective early-retrieval performance may not provide a statistically meaningful advantage for prospective screening. Nevertheless, the present finding should be interpreted cautiously, as it pertains to a low-data regime for this specific protein, although strong test-set screening power for untuned target-specific models has been reported for several other targets [42, 48]. Whether this observation generalizes more broadly, for example, to model or pipeline selection across more diverse targets, screening strategies or larger data sets, remains to be investigated in future studies.

Regarding classification power (Fig. 4B; Table S3, Supporting Information), optimizing both hyperparameters and decision thresholds on the training data enabled the tuned models to distinguish between classes on the held-out test set more effectively (balanced accuracy = 0.8573 ± 0.1286, p-value < 0.05) than the untuned models (balanced accuracy = 0.6705 ± 0.1277). In contrast, balancing the training set did not lead to a substantial improvement in test-set balanced accuracy: differences between the default and oversampling-only categories, as well as between tuning-only and tuning-plus-oversampling, were not statistically significant (p-value > 0.05). These results indicate that minority-class oversampling alone did not significantly enhance predictive performance on unseen data, contrary to the common expectation that it should alleviate class-imbalance effects in binary classification. Instead, improvements were primarily driven by model tuning, encompassing both hyperparameter and decision-threshold optimization. Consistent with the screening-power analysis above, artificially balancing the training set did not improve either screening or classification outcomes for this target, whereas appropriate model tuning did enhance the models’ ability to correctly classify compounds.

## Conclusions

In this study, we assembled the most comprehensive and up-to-date data set of CBS inhibitors, integrating experimental information from PubChem, ChEMBL, the scientific literature, and three chemical patents. Incorporating data from publications and patents substantially enriched the active population beyond what could be obtained from commonly used public repositories alone, underscoring the importance of mining diverse sources, particularly for targets such as CBS that remain relatively underexplored. To expand the inactive population, we followed recent recommendations and incorporated DCM compounds as assumed inactives, retaining only those whose physicochemical properties matched the ranges observed for the true actives, thereby limiting potential bias and ensuring a realistic and balanced chemical space representation. We next applied a rigorous stratified data-splitting strategy, partitioning the full data set into five folds that were structurally independent while remaining comparably challenging. This design minimized analogue bias across folds and supported reliable model assessment, further demonstrating the effectiveness of recently recommended UMAP-based clustering combined with similarity-controlled splitting procedures for constructing robust training-test partitions in VS studies.

Across the five training-test splits of this five-fold CV framework, we trained and evaluated a series of CBS-specific AI binary classification models for CBS inhibitor prediction, systematically investigating multiple modeling choices, including default training, hyperparameter tuning only, minority-class oversampling only, and the combination of tuning and oversampling. To our knowledge, this represents the first development of target-specific AI models for this protein. In parallel, we benchmarked a diverse panel of existing structure-based VS tools on the same folds, including: (i) baseline pipelines based on Smina docking, RF-Score-VS rescoring, and GNINA 1.3 docking with CNN scoring; (ii) SOTA end-to-end DL docking tools such as DeepDock, Interformer, SurfDock, KarmaDock; and (iii) co-folding approaches including Boltz-2, Boltz-2x and AlphaFold 3. Benchmarking such a broad range of established and SOTA tools ensures a comprehensive, diverse, and fair comparison framework that captures both conventional and the most recent methodological advances in the field [63], particularly those driven by progress in AI [64]. This work therefore also constitutes the first systematic evaluation of these modern tools on CBS, a target that is weakly represented, if present at all, in their training data, enabling us to assess their ability to extrapolate to previously under-represented targets in a VS context.

Our results revealed several key findings. First, across all five folds, and also when folds were analyzed individually, the CBS-specific AI pipelines consistently outperformed the 16 reference VS pipelines, achieving significantly higher NEF1% values across all categories of existing tools. This observation aligns with prior studies emphasizing the strong screening capability of target-specific AI scoring functions in structure-based VS. Second, generic SOTA approaches, including end-to-end DL docking tools and co-folding methods, showed clear limitations when applied to a target that is poorly represented in their training distributions, particularly in this low-data regime. This highlights ongoing challenges of successfully applying such models to underexplored targets in VS, complementing similar observations previously reported for other drug-design tasks, and indicating that strong overall benchmark performance does not necessarily guarantee reliable prioritization for these targets. Third, target-specific models trained with hyperparameters optimized for early retrieval, or on artificially balanced training sets, did not exhibit substantially improved screening performance compared with models trained on the original imbalanced data without tuning. In contrast, optimizing both hyperparameters and decision thresholds markedly improved classification performance on unseen test data. Minority-class oversampling alone did not significantly enhance predictive performance, contrary to common expectations; instead, performance gains were primarily driven by comprehensive model tuning. These findings provide practical guidance for researchers developing target-specific AI models, indicating that decision-threshold and hyperparameter optimization may be more impactful than data set balancing, depending on whether the primary goal is classification accuracy or VS enrichment.

All input and output data, trained models and source code from this work are freely available through our GitHub repository and Zenodo archive, supporting open science and enabling the community to build upon these resources. Given the still-emerging state of CBS research, these openly accessible assets may facilitate future studies aimed at discovering and optimizing CBS inhibitors (e.g., through prospective VS campaigns on ultra-large chemical libraries), further highlighting the value and practical impact of the present work.

## Methods

### Data retrieval, data curation and stratified data split

#### Data retrieval and curation

Raw experimental data reporting human CBS activity were retrieved from PubChem and ChEMBL v.36 (as of January 2026) [18, 19], using the UniProt accession code P35520, following a previously published data-collection protocol [42]. For each compound evaluated in vitro (in single-protein assays), the retrieved records included a canonical SMILES string, molecular ID(s), activity phenotype(s) and corresponding activity label(s) (active/inactive), associated bioassay(s) (assay ID(s) and classification as primary or confirmatory), and reported activity concentration(s) when available. Detailed metadata describing the bioassays were also collected. Only data entries explicitly reporting CBS inhibitory activity were retained for further analysis. To expand the data set beyond these two public repositories, additional compounds were extracted from a recent review on CBS inhibitors [16] and three chemical patents (WO2010072807A2, WO2010072807A3 and WO2013068592A1) [39–41]. The resulting raw data set was then processed using a systematic data-curation workflow, as described in our recent study [21]. This procedure comprised three main steps: (i) removal of ambiguous entries and duplicate records, (ii) assignment of a unified activity label and potency value to each compound, and (iii) exclusion of potential false positives arising from assay interference. Specifically, salt counterions and small molecular fragments were removed from each SMILES string using the ‘SaltRemover’ module from ‘rdkit.Chem’, followed by SMILES canonicalization via the ‘CanonSmiles’ function and validation of each SMILES using ‘AddMoleculeColumnToFrame’ from ‘rdkit.Chem.PandasTools’ (RDKit v.2025.03.2). For activity labeling, an IC_50_ threshold of 10 μM was selected as a universal cutoff to distinguish experimental hits from non-hits, consistent with PubChem annotation practices and commonly adopted to define strong CBS inhibitors [14]. This stringent criterion was chosen to ensure the positive class consisted of highly potent inhibitors, particularly important for subsequent model training. While this threshold reduced the number of actives, it yielded a more challenging and realistic data set that better reflected real-world screening scenarios, where true positives are typically scarce. Additional details regarding the data-curation procedure are provided elsewhere [21].

In addition to experimentally validated actives and inactives collected from the sources described above, we further expanded the negative class by incorporating compounds from the DCM space. This approach was motivated by recent findings demonstrating that AI models trained using DCM compounds as assumed inactives closely reproduce the performance of models trained on experimentally confirmed non-binders, thereby providing a practical strategy for targets with limited inactivity data [27]. The DCM library was obtained directly from the ChemDiv webserver (accessed January 2026) and comprised 18,513 compounds [65, 66]. SMILES representations were retrieved for all compounds and processed using the RDKit pipeline applied to the true actives and true inactives. The ‘mol’ objects obtained from all processed SMILES (via ‘AddMoleculeColumnToFrame’ from ‘rdkit.Chem.PandasTools’, RDKit v.2025.03.2) were subsequently used to calculate eight physicochemical properties: MW (‘ExactMolWt’, ‘rdkit.Chem.Descriptors’), atom count and bond count (‘GetNumAtoms’ and ‘GetNumBonds’, ‘rdkit.Chem.rdchem’), ClogP (‘MolLogP’, ‘rdkit.Chem.Crippen’), hydrogen-bond acceptor and donor counts (‘NumHAcceptors’ and ‘NumHDonors’, ‘rdkit.Chem.Lipinski’), rotatable bond count (‘CalcNumRotatableBonds’, ‘rdkit.Chem.rdMolDescriptors’), and TPSA (‘CalcTPSA’, ‘rdkit.Chem.rdMolDescriptors’). Only DCM compounds for which all eight physicochemical properties fell within the corresponding ranges of the true actives were retained, thereby ensuring that only property-matched “assumed inactives” were included in the study.

To ensure consistent processing and preparation across all compounds in the data set, including true actives, true inactives, and remaining DCM compounds, an identical preparation workflow was applied. This strategy ensured that all ligands provided to docking pipelines were generated through the same standardized preparation procedure, regardless of their source, thereby minimizing preparation-induced biases (exceptions applied only to methods that directly operate on ligand SMILES strings and/or protein FASTA sequences as input, such as co-folding approaches). Three-dimensional structures were generated from the previously processed SMILES representations of all molecules, and the resulting structures were protonated at physiological pH (7.4) using Open Babel (v.3.1.0) [67], in accordance with practices described in recent protocols and studies [21, 42]. All ligand structures were saved in sdf and mol2 formats and used for molecular docking.

#### Stratified data split

To distribute the full data set comprising 43 true actives and 10,402 inactives (195 true inactives and 10207 DCM compounds) into five CV folds, we adopted a clustering-based splitting strategy built upon dimensionality reduction with UMAP. All compounds were first represented using 1,024-bit Morgan fingerprints with a radius of 2 and were subsequently embedded into a two-dimensional space using UMAP (v.0.5.7) with the Jaccard distance metric. The resulting low-dimensional representations of active and inactive compounds were then clustered separately by class using agglomerative hierarchical clustering (actives were clustered only with actives and inactives only with inactives). Clustering was performed with the ‘AgglomerativeClustering’ implementation from the ‘sklearn.cluster’ module (v.1.5.1), employing Ward linkage and Euclidean distance. This UMAP-based clustering strategy follows recent studies demonstrating that it produces higher-quality and more challenging molecular splits than traditional approaches such as Butina clustering or scaffold-based splits, enabling more realistic model evaluation [28, 44]. To optimize the clustering procedure, two UMAP hyperparameters (‘n_neighbors’ and ‘min_dist’) and one agglomerative clustering hyperparameter (‘n_clusters’) were tuned using Optuna (v.4.4.0) with a Tree-structured Parzen Estimator (TPE) sampler over 100 optimization trials. The silhouette score was used as the optimization objective. This hyperparameter optimization was conducted independently for the active and inactive subsets.

Following clustering, entire clusters were randomly assigned to one of five folds under the following constraints: (i) each cluster was assigned in its entirety to a single fold, ensuring that no members of the same cluster appeared across different folds, thereby preventing data leakage during five-fold CV and preserving clear structural separation between training and test data; (ii) the maximum pairwise Tanimoto similarity (Morgan fingerprints, 2048 bits, radius 2) between compounds of the same class across different folds did not exceed 0.70; (iii) the eight calculated physicochemical properties of actives and inactives substantially overlapped within each fold; (iv) the ratio of active to inactive compounds was maintained consistently low across all folds; and (v) the total number of compounds was approximately balanced among folds.

### Molecular docking using Smina (v.2019–10-15) and GNINA 1.3

The protein template selected for molecular docking in this study was PDB ID 7QGT. This structure met several key criteria: (i) it is a human CBS complex co-crystallized with the AOAA inhibitor; (ii) it contains no mutations and shows 100% sequence identity with the UniProt reference sequence (P35520), as confirmed by sequence alignment using the ‘Align’ tool with the Clustal Omega program on the UniProt webserver [68]; (iii) it exhibits acceptable crystallographic quality, with a resolution of 2.69 Å, an R-free value of 0.242, an R-work value of 0.208, and an RSRZ (Real Space R-factor Z-score) outlier proportion (i.e., fraction of residues with poor electron density fit) of 1.1%; and (iv) it displays no Ramachandran outlier and a low proportion of side-chain outliers (1.2%). Protein preparation was performed using the ‘DockPrep’ tool in ChimeraX (v.1.8), with parameters set according to the recommendations of dos Santos et al. [69]. Hydrogen atoms and partial charges were added based on the atom types of hydrogen-bearing heavy atoms, and incomplete side chains were rebuilt using the Dunbrack rotamer library. The final prepared protein structure was saved in both pdb and mol2 formats for subsequent docking studies.

Molecular docking was performed using Smina (v.2019–10-15) and GNINA 1.3 [29, 38], employing the prepared protein template in mol2 format and the prepared ligand structures in sdf format, as described above. The binding site was defined using a cubic grid box with a volume of 27,000 Å^3^ (30 Å × 30 Å × 30 Å), centered on the centroid of the co-crystallized ligand AOAA. The AOAA structure, retrieved directly from the RCSB PDB in sdf, was used to define the docking region via the ‘autobox_ligand’ option. Docking calculations were conducted with an ‘exhaustiveness’ value of 8 (default setting), and the number of output binding modes (‘num_modes’) was set to 1. A fixed random seed was specified to ensure reproducibility across docking runs. For each compound, one docked pose per docking program was generated and saved in sdf format. Using these settings, the co-crystallized ligand AOAA yielded a Smina-redocked pose with an RMSD (root-mean-square deviation) of 1.48 Å relative to the ground-truth pose, satisfying all PoseBusters physicochemical quality criteria [56].

Smina-generated docking poses were first scored using its built-in empirical scoring function [29]. They were then rescored using RF-Score-VS v.2 [37], following the steps outlined in a recent protocol [42]. In contrast, docking poses generated by GNINA 1.3 were evaluated using its CNN-based scoring function (compounds ranked by “CNNscore”) [38], a SOTA DL approach, and were therefore not subjected to any additional rescoring by other AI-based scoring functions.

### End-to-end DL-based molecular docking

#### DeepDock

The previously prepared protein structure (PDB ID 7QGT, pdb format) and the co-crystallized ligand AOAA (mol2 format) were used to generate a polygonal mesh representing the molecular surface of the binding site, defined as all protein atoms within 10 Å of any atom of the crystal ligand. This surface mesh (ply format), along with each prepared compound structure (mol2 format), was then used to generate a single optimized ligand pose per compound, together with its corresponding negative potential *U*_*(x)*_ (reported as the “fun” score in the output). Molecules within each fold were subsequently ranked according to these scores, from which screening power was evaluated. All DeepDock simulations were performed using the official codebase, with parameters kept at their default values, as provided in the DeepDock GitHub repository (https://github.com/OptiMaL-PSE-Lab/DeepDock; accessed August 2025).

#### Interformer

The complete protein–ligand complex (PDB ID 7QGT, pdb format) and the co-crystallized ligand AOAA (sdf format) were first used to define the binding pocket, consisting of all protein atoms whose distance to any ligand atom was < 7 Å. Each prepared compound (sdf format) of our data set was then energy-minimized using the universal force field (UFF). Using the defined pocket and energy-minimized ligands, 20 poses per compound were generated along with their corresponding “Pose Energy.” These poses were subsequently input, without further modification, into the Interformer “Affinity and PoseScore” layer to predict the pIC_50_ and the confidence “Pose Score.” Pose selection (one pose per docked molecule) was carried out based on either the “Pose Energy” (retaining the lowest-energy pose) or the “Pose Score” (retaining the highest-scored pose), after which ligands were ranked according to their predicted pIC_50_ values. All parameters were retained at their default values. The source code was obtained directly from the Interformer GitHub repository (https://github.com/tencent-ailab/Interformer; accessed August 2025).

#### SurfDock

The previously prepared protein structure (PDB ID 7QGT, pdb format) and the co-crystallized ligand AOAA (sdf format) were used to define the binding pocket as all protein residues within 8 Å of any ligand atom. For each prepared compound in our data set (sdf format), SurfDock generated 40 poses together with their corresponding “pose prediction confidence” scores. These poses were subsequently rescored to obtain a “screen confidence” score, which was used to rank the molecules. The screening workflow was executed exactly as implemented in the bash script ‘screen_pipeline.sh’ provided in the SurfDock GitHub repository (https://github.com/CAODH/SurfDock/blob/master/bash_scripts/test_scripts/screen_pipeline.sh; accessed February 2026).

#### KarmaDock

The previously prepared protein structure (PDB ID 7QGT, pdb format) and the co-crystallized ligand AOAA (mol2 format) were first used to define the binding pocket, encompassing all protein residues within 12 Å of the crystal ligand atoms. Each compound, represented as a canonical SMILES string generated by the RDKit (cf. “Data retrieval and curation”), was then docked into this pocket to produce a single pose and its corresponding “KarmaDock raw” docking score. This initial docking pose was subsequently post-processed by either force-field optimization (MMFF94) or an RDKit-based conformational alignment procedure. In the latter, a chemically ideal RDKit-generated conformation (with standard bond lengths, angles and torsions) was mapped onto the fixed predicted docking pose using the Kabsch algorithm. The transformed RDKit structure then replaced the original pose while preserving its binding orientation and without reference to any external experimental structure, thereby correcting local geometric inconsistencies. This yielded two additional processed poses with “KarmaDock-FF” and “KarmaDock-aligned” docking scores. Molecules within each fold were ranked separately according to each of these three scores, and screening power was evaluated for each ranking scenario. The KarmaDock source code, with all default parameters, was obtained from GitHub (https://github.com/schrojunzhang/KarmaDock; accessed November 2025).

### Protein–ligand complex generation using co-folding approaches

#### Boltz-2 and Boltz-2x

Input files for Boltz-2 and Boltz-2x were prepared in yaml format, including the protein FASTA sequence (PDB ID 7QGT) and the canonical SMILES string for each compound of our data set. For Boltz-2x, physical steering potentials were enabled to enhance physicochemical validity during pose sampling. One binding pose (in pdb format) was generated per screened molecule. All other parameters were kept at their default settings, with the source code obtained from GitHub (https://github.com/jwohlwend/boltz; accessed June 2025, v.2.1.1). According to the recommendations of the Boltz authors [70], the “affinity_probability_binary,” representing the predicted probability that a ligand is a binder, was used to rank screened molecules within each fold, from which screening power was evaluated.

#### AlphaFold 3

Input files for AlphaFold 3 were prepared in json format, including the protein FASTA sequence (according to PDB ID 7QGT) together with its multiple sequence alignment (MSA) and structural templates, as well as the canonical SMILES string for each compound. In this study, we followed the staged pipeline execution procedure described in the official documentation (https://github.com/google-deepmind/alphafold3/blob/main/docs/performance.md; accessed February 2025). Specifically, in the first stage, MSAs and templates for the protein were precomputed using the ‘–run_inference = false’ flag. The resulting ‘unpairedMsa’, ‘pairedMsa’ and ‘templates’ fields were then combined with the ligand canonical SMILES string into a single json input file. This file was subsequently used directly for the inference stage with the ‘–run_data_pipeline = false’ flag to reduce computational cost. Genetic databases were downloaded in June 2025, and model parameters were obtained from Google in January 2026. AlphaFold 3 (v. 3.0.1) was executed within a Singularity environment; the container was built in May 2025. For each compound, the number of generated complexes (‘num_diffusion_samples’) was kept at its default value of 5, and the complex with the highest “ranking score” was retained as the representative pose, following official AlphaFold recommendations (https://github.com/google-deepmind/alphafold3/blob/main/docs/output.md; accessed February 2025). Compounds were ranked using one of the reported confidence metrics, including “pTM” (predicted TM-score), “ipTM” (predicted interface TM-score), the overall “ranking score,” and “min-iPAE” (minimum pairwise predicted aligned error between protein and ligand tokens), as practiced in a recent study [54].

### CBS-specific AI scoring functions for inhibitor prediction

Following practices in the literature [21, 30, 42], PLEC fingerprints were extracted from Smina docking poses using our in-house Python code (available along with a detailed tutorial at https://github.com/caominhtr/CBS/tree/main/tools), and were next provided as input to four AI algorithms (RF, XGBoost, SVM, MLP) to train our binary classifiers for CBS inhibitors. The models were implemented using the ‘RandomForestClassifier’, ‘SVC’ and ‘MLPClassifier’ functions from the ‘sklearn.ensemble’, ‘sklearn.svm’ and ‘sklearn.neural_network’ modules (‘scikit-learn’ v.1.5.1) for RF, SVM and MLP, respectively; and the ‘XGBClassifier’ function from the ‘xgboost’ library (v.2.1.1) for XGBoost.

Within our five-fold CV setup (cf. “Computational workflow”), model training explored different strategies, including hyperparameter tuning followed by decision threshold optimization, and/or oversampling of the minority class (actives), representing the investigated modeling choices. Hyperparameter and decision threshold optimization was performed using Optuna (v.4.4.0) with 100 trials and a TPE sampler. First, screening performance was maximized by optimizing the average NEF1% across a four-fold internal CV on the training data (comprising four folds with their original composition preserved) to select optimal hyperparameters. Subsequently, classification performance was maximized by tuning the decision threshold for models with previously optimized hyperparameters, using the average balanced accuracy from the same four-fold internal CV as the objective metric. The search spaces and resulting optimal hyperparameters are reported in Tables S4 and S5 (Supporting Information). To address class imbalance, oversampling of the minority class was performed using the ‘RandomOverSampler’ function from the ‘imbalanced-learn’ library (v.0.12.3), with the sampling strategy set to “auto” to equalize class sizes after resampling. A fixed random state of 42 ensured reproducibility.

Final tuned models were retrained on the complete training set, either with or without oversampling according to the modeling choice, and were saved as pickle files for future inference. All source code and trained models are publicly available in our GitHub repository and on Zenodo (cf. “Data availability”).

### Evaluation of screening and classification power

For all evaluated tools in our benchmarking, including our CBS-specific AI models, screening power was quantified using the NEF1% on each fold. NEF1% is defined as the ratio between the EF1% achieved by a given tool on a test fold and the maximal EF1% attainable on that fold, thereby reflecting the proportion of the maximal enrichment capacity recovered within the top 1% of ranked instances. This normalization enables fair comparison across different VS tools and across folds with varying class distributions. In our five-fold setup, the number of active compounds in each fold was lower than 1% of the fold size (cf. Table 1). Consequently, the maximal EF1% was obtained when all actives were retrieved within the top 1% of ranked molecules, corresponding to a maximal EF1% of 100 for all folds. The NEF1% in this study was therefore calculated as follows [42, 71, 72]:

$$NEF1\%=\frac{EF1\%}{maxEF1\%}=\frac{\frac{{n}_{a}}{n}\times 100}{100}=\frac{{n}_{a}}{n}$$

where ${n}_{a}$ denotes the number of active compounds retrieved within the top 1% of the ranked list, and n denotes the total number of active compounds in the corresponding fold.

Only for our CBS-specific binary classifiers, balanced accuracy was reported to account for the pronounced class imbalance in our data set [49]. For each model, a confusion matrix was first computed using either a fixed decision threshold of 0.50 (for default models) or an optimized threshold determined via internal four-fold CV on the training data (for tuned models). Balanced accuracy was subsequently calculated as follows:

$$Balanced accuracy=\frac{1}{2}\left(Recall+Specificity\right)=\frac{1}{2}\left(\frac{TP}{P}+\frac{TN}{N}\right)$$

Here, TP and TN denote the numbers of true positives and true negatives, respectively, while P and N represent the total numbers of positive and negative instances in the corresponding fold.

## Supplementary Information

Supplementary Material 1.

## Data Availability

All relevant source code, data sets and results reported in this article are available free of charge in our GitHub repository (https://github.com/caominhtr/CBS, please read the README file for more information) and on Zenodo (https://zenodo.org/records/18743776), ensuring long-term accessibility.

## Abbreviations

AI: Artificial intelligence
AOAA: Aminooxyacetic acid
CBS: Cystathionine β-synthase
CV: Cross-validation
DCM: Dark chemical matter
DL: Deep learning
IC_50_: Half-maximal inhibitory concentration
ID: Identifier
ML: Machine learning
MLP: Multilayer perceptron
MW: Molecular weight
NEF1%: Normalized enrichment factor of true actives at 1%
PDB: Protein data bank
PLEC: Protein–ligand extended connectivity
RF: Random forest
RMSD: Root-mean-square deviation
RSRZ: Real Space R-factor Z-score
SOTA: State-of-the-art
SVM: Support vector machine
TPE: Tree-structured Parzen Estimator
TPSA: Topological polar surface area
UFF: Universal force field
UMAP: Uniform manifold approximation and projection
VS: Virtual screening
XGBoost: Extreme gradient boosting

## References

[1] Irving C, Basu A, Richmond S et al (2008) Twenty-year trends in prevalence and survival of Down syndrome. Eur J Hum Genet 16:1336–1340 10.1038/ejhg.2008.122 18596694

[2] Zou C-G, Banerjee R (2005) Homocysteine and redox signaling. Antioxid Redox Signal 7:547–559 10.1089/ars.2005.7.547 15890000

[3] Chen X, Jhee K-H, Kruger WD (2004) Production of the neuromodulator H_2_S by cystathionine β-synthase via the condensation of cysteine and homocysteine. J Biol Chem 279:52082–52086 10.1074/jbc.C400481200 15520012

[4] Jhee K-H, McPhie P, Miles EW (2000) Domain architecture of the heme-independent yeast cystathionine β-synthase provides insights into mechanisms of catalysis and regulation. Biochemistry 39:10548–10556 10.1021/bi001020g 10956046

[5] McCorvie TJ, Kopec J, Hyung S-J et al (2014) Inter-domain communication of human cystathionine β-synthase. J Biol Chem 289:36018–36030 10.1074/jbc.M114.610782 PMC4276868 25336647

[6] London J, Ndiaye FK, Bui LC et al (2019) Alterations in the serotonin and dopamine pathways by cystathionine beta synthase overexpression in murine brain. Mol Neurobiol 56:3958–3971 10.1007/s12035-018-1323-2 30238388

[7] Robert K, Vialard F, Thiery E et al (2003) Expression of the cystathionine β synthase (CBS) gene during mouse development and immunolocalization in adult brain. J Histochem Cytochem 51:363–371 10.1177/002215540305100311 12588964

[8] Ichinohe A, Kanaumi T, Takashima S et al (2005) Cystathionine β-synthase is enriched in the brains of Down’s patients. Biochem Biophys Res Commun 338:1547–1550 10.1016/j.bbrc.2005.10.118 16274669

[9] Chadefaux B, Rethoré MO, Raoul O et al (1985) Cystathionine beta synthase: gene dosage effect in trisomy 21. Biochem Biophys Res Commun 128:40–44 10.1016/0006-291x(85)91641-9 3157380

[10] Kamoun P, Belardinelli M, Chabli A et al (2003) Endogenous hydrogen sulfide overproduction in Down syndrome. Am J Med Genet A 116A:310–311 10.1002/ajmg.a.10847 12503113

[11] Kamoun P (2004) Endogenous production of hydrogen sulfide in mammals. Amino Acids 26:243–254 10.1007/s00726-004-0072-x 15221504

[12] Qu K, Lee SW, Bian JS et al (2008) Hydrogen sulfide: neurochemistry and neurobiology. Neurochem Int 52:155–165 10.1016/j.neuint.2007.05.016 17629356

[13] Hu L-F, Lu M, Hon Wong PT, Bian J-S (2011) Hydrogen sulfide: neurophysiology and neuropathology. Antioxid Redox Signal 15:405–419 10.1089/ars.2010.3517 20812864

[14] Niu W, Wu P, Chen F et al (2017) Discovery of selective cystathionine β-synthase inhibitors by high-throughput screening with a fluorescent thiol probe. MedChemComm 8:198–201 10.1039/c6md00493h PMC6072345 30108705

[15] Petrosino M, Zuhra K, Kopec J et al (2022) H2S biogenesis by cystathionine beta-synthase: mechanism of inhibition by aminooxyacetic acid and unexpected role of serine. Cell Mol Life Sci 79:438 10.1007/s00018-022-04479-9 PMC9304066 35864237

[16] Zuhra K, Augsburger F, Majtan T, Szabo C (2020) Cystathionine-β-synthase: molecular regulation and pharmacological inhibition. Biomolecules 10:697 10.3390/biom10050697 PMC7277093 32365821

[17] Ascenção K, Szabo C (2022) Emerging roles of cystathionine β-synthase in various forms of cancer. Redox Biol 53:102331 10.1016/j.redox.2022.102331 PMC9168780 35618601

[18] Kim S, Chen J, Cheng T et al (2025) PubChem 2025 update. Nucleic Acids Res 53:D1516–D1525 10.1093/nar/gkae1059 PMC11701573 39558165

[19] Gaulton A, Bellis LJ, Bento AP et al (2012) ChEMBL: a large-scale bioactivity database for drug discovery. Nucleic Acids Res 40:D1100–D1107 10.1093/nar/gkr777 PMC3245175 21948594

[20] Chua HN, Jasser MB, Issa B, Wong RTK (2025) Differentiating Artificial Intelligence, Machine Learning, Deep Learning, and Data Mining. 2025 IEEE International Conference on Automatic Control and Intelligent Systems (I2CACIS). IEEE, Kuala Lumpur, Malaysia, pp 250–255

[21] Tran-Nguyen V-K, Randriharimanamizara UF, Taboureau O (2025) HERGAI: an artificial intelligence tool for structure-based prediction of hERG inhibitors. J Cheminform 17:110 10.1186/s13321-025-01063-8 PMC12291323 40708034

[22] Gómez-Sacristán P, Simeon S, Tran-Nguyen V-K et al (2025) Inactive-enriched machine-learning models exploiting patent data improve structure-based virtual screening for PDL1 dimerizers. J Adv Res 67:185–196 10.1016/j.jare.2024.01.024 PMC11725107 38280715

[23] Caba K, Tran-Nguyen V-K, Rahman T, Ballester PJ (2024) Comprehensive machine learning boosts structure-based virtual screening for PARP1 inhibitors. J Cheminform 16:40 10.1186/s13321-024-00832-1 PMC10999096 38582911

[24] Castillo Tarazona MY, Miscione GP 2026 Enhancing structure-based virtual screening of MDM2–p53 inhibitors: a benchmark of machine learning vs. traditional docking scoring functions. Front Drug Discov 5 1731262.

[25] Serghini A, Zhou Y, Myung Y et al (2026) KinCSM-RTK: machine learning-based screening of receptor tyrosine kinase inhibitors in drug discovery. J Chem Inf Model 66:61–73 10.1021/acs.jcim.5c01677 41423943

[26] Tran-Nguyen V-K, Camproux A-C, Taboureau O (2024) ClassyPose: A Machine-Learning Classification Model for Ligand Pose Selection Applied to Virtual Screening in Drug Discovery. Adv Intell Syst 6:2400238

[27] Victoria-Muñoz F, Menke J, Sanchez-Cruz N, Koch O (2025) Efficient decoy selection to improve virtual screening using machine learning models. J Cheminform 17:165 10.1186/s13321-025-01107-z PMC12577370 41168868

[28] Guo Q, Hernandez-Hernandez S, Ballester PJ (2025) UMAP-based clustering split for rigorous evaluation of AI models for virtual screening on cancer cell lines*. J Cheminform 17:94 10.1186/s13321-025-01039-8 PMC12153141 40495205

[29] Koes DR, Baumgartner MP, Camacho CJ (2013) Lessons learned in empirical scoring with smina from the CSAR 2011 benchmarking exercise. J Chem Inf Model 53:1893–1904 10.1021/ci300604z PMC3726561 23379370

[30] Wójcikowski M, Kukiełka M, Stepniewska-Dziubinska MM et al (2019) Development of a protein–ligand extended connectivity (PLEC) fingerprint and its application for binding affinity predictions. Bioinformatics 35:1334–1341 10.1093/bioinformatics/bty757 PMC6477977 30202917

[31] Méndez-Lucio O, Ahmad M, Del Rio-Chanona EA, Wegner JK (2021) A geometric deep learning approach to predict binding conformations of bioactive molecules. Nat Mach Intell 3:1033–1039

[32] Lai H, Wang L, Qian R et al (2024) Interformer: an interaction-aware model for protein-ligand docking and affinity prediction. Nat Commun 15:10223 10.1038/s41467-024-54440-6 PMC11589619 39587070

[33] Cao D, Chen M, Zhang R et al (2025) SurfDock is a surface-informed diffusion generative model for reliable and accurate protein–ligand complex prediction. Nat Methods 22:310–322 10.1038/s41592-024-02516-y 39604569

[34] Zhang X, Zhang O, Shen C et al (2023) Efficient and accurate large library ligand docking with KarmaDock. Nat Comput Sci 3:789–804 10.1038/s43588-023-00511-5 38177786

[35] Passaro S, Corso G, Wohlwend J et al (2025) Boltz-2: Towards Accurate and Efficient Binding Affinity Prediction. Preprint at bioRxiv. 10.1101/2025.06.14.659707

[36] Abramson J, Adler J, Dunger J et al (2024) Accurate structure prediction of biomolecular interactions with AlphaFold 3. Nature 630:493–500 10.1038/s41586-024-07487-w PMC11168924 38718835

[37] Wójcikowski M, Ballester PJ, Siedlecki P (2017) Performance of machine-learning scoring functions in structure-based virtual screening. Sci Rep 7:46710 10.1038/srep46710 PMC5404222 28440302

[38] McNutt AT, Li Y, Meli R et al (2025) GNINA 1.3: the next increment in molecular docking with deep learning. J Cheminform 17:28 10.1186/s13321-025-00973-x PMC11874439 40025560

[39] Blehaut H, Bellamy F, Matt C, Giraud S, Charre D (2010) Inhibitors of cystathionine beta synthase to reduce the neurotoxic overproduction of endogenous hydrogen sulfide. WO2010072807A2, 1 Jul 2010.

[40] Blehaut H, Bellamy F, Matt C, Giraud S, Charre D (2010) Inhibitors of cystathionine beta synthase for preventing or treating neurotoxic or cognitive disorders. WO2010072807A3

[41] Charre D, Blehaut H, Bellamy F 2013 Inhibitors of cystathionine beta synthase to reduce the neurotoxic overproduction of endogenous hydrogen sulfide. WO2013068592A1

[42] Tran-Nguyen V-K, Junaid M, Simeon S, Ballester PJ (2023) A practical guide to machine-learning scoring for structure-based virtual screening. Nat Protoc 18:3460–3511 10.1038/s41596-023-00885-w 37845361

[43] Tran-Nguyen V-K, Jacquemard C, Rognan D (2020) LIT-PCBA: an unbiased data set for machine learning and virtual screening. J Chem Inf Model 60:4263–4273 10.1021/acs.jcim.0c00155 32282202

[44] Hernández-Hernández S, Ballester PJ (2023) On the best way to cluster NCI-60 molecules. Biomolecules 13:498 10.3390/biom13030498 PMC10046274 36979433

[45] Camilleri F, Wenda JM, Pecoraro-Mercier C et al (2024) Cell painting and chemical structure read-across can complement each other for rat acute oral toxicity prediction in chemical early derisking. Chem Res Toxicol 37:1851–1866 10.1021/acs.chemrestox.4c00169 39513413

[46] Arab I, Laukens K, Bittremieux W (2024) Semisupervised learning to boost hERG, Nav1.5, and Cav1.2 cardiac ion channel toxicity prediction by mining a large unlabeled small molecule data set. J Chem Inf Model 64:6410–6420 10.1021/acs.jcim.4c01102 39110924

[47] Kumar A (2011) Chemical similarity methods- a tutorial review. Chem Educator 16:46–50

[48] Wellnitz J, Jain S, Hochuli JE et al (2025) One size does not fit all: revising traditional paradigms for assessing accuracy of QSAR models used for virtual screening. J Cheminform 17:7 10.1186/s13321-025-00948-y PMC11740363 39819357

[49] Thölke P, Mantilla-Ramos Y-J, Abdelhedi H et al (2023) Class imbalance should not throw you off balance: choosing the right classifiers and performance metrics for brain decoding with imbalanced data. Neuroimage 277:120253 10.1016/j.neuroimage.2023.120253 37385392

[50] Ballester PJ (2019) Selecting machine-learning scoring functions for structure-based virtual screening. Drug Discov Today Technol 32–33:81–87 10.1016/j.ddtec.2020.09.001 33386098

[51] Ghislat G, Rahman T, Ballester PJ (2021) Recent progress on the prospective application of machine learning to structure-based virtual screening. Curr Opin Chem Biol 65:28–34 10.1016/j.cbpa.2021.04.009 34052776

[52] Truchon J-F, Bayly CI (2007) Evaluating virtual screening methods: good and bad metrics for the “early recognition” problem. J Chem Inf Model 47:488–508 10.1021/ci600426e 17288412

[53] Li H, Leung K-S, Wong M-H, Ballester PJ (2016) Correcting the impact of docking pose generation error on binding affinity prediction. BMC Bioinformatics 17:308 10.1186/s12859-016-1169-4 PMC5046193 28185549

[54] Shen C, Zhang X, Gu S et al (2026) Unlocking the application potential of AlphaFold3-like approaches in virtual screening. Chem Sci 17:2858–2879 10.1039/d5sc06481c PMC12704515 41403459

[55] Li Y, Yi J, Li H et al (2025) Decoding the limits of deep learning in molecular docking for drug discovery. Chem Sci 16:17374–17390 10.1039/d5sc05395a PMC12401186 40901622

[56] Buttenschoen M, Morris GM, Deane CM (2024) PoseBusters: AI-based docking methods fail to generate physically valid poses or generalise to novel sequences. Chem Sci 15:3130–3139 10.1039/d3sc04185a PMC10901501 38425520

[57] Mastropietro A, Pasculli G, Bajorath J (2023) Learning characteristics of graph neural networks predicting protein–ligand affinities. Nat Mach Intell 5:1427–1436

[58] Jain AN, Cleves AE, Walters WP 2024 Deep-Learning Based Docking Methods: Fair Comparisons to Conventional Docking Workflows. Preprint at arXiv. 10.48550/arXiv.2412.02889.

[59] Truong C-M, Ballester PJ, Taboureau O, Tran-Nguyen V-K (2026) NextTopDocker: the largest-to-date docking power benchmark reveals that deep learning performs generally much worse than logistic regression models. Preprint at ChemRxiv. 10.26434/chemrxiv.10001776/v2.

[60] Masters MR, Mahmoud AH, Lill MA (2025) Investigating whether deep learning models for co-folding learn the physics of protein-ligand interactions. Nat Commun 16:8854 10.1038/s41467-025-63947-5 PMC12501370 41053181

[61] Nittinger E, Yoluk Ö, Tibo A et al (2025) Co-folding, the future of docking – prediction of allosteric and orthosteric ligands. Artif Intell Life Sci 8:100136

[62] Grieswelle M, Homberg S, Janssen P, et al (2026) A new benchmark for deep learning based affinity prediction: Solving the inter-protein scoring noise problem. Preprint at ChemRxiv. 10.26434/chemrxiv-2025-sf3cs/v2.

[63] Tran-Nguyen V-K, Ballester PJ (2023) Beware of simple methods for structure-based virtual screening: the critical importance of broader comparisons. J Chem Inf Model 63:1401–1405 10.1021/acs.jcim.3c00218 PMC10015451 36848585

[64] Mroz AM, Basford AR, Hastedt F et al (2025) Cross-disciplinary perspectives on the potential for artificial intelligence across chemistry. Chem Soc Rev 54:5433–5469 10.1039/d5cs00146c PMC12024683 40278836

[65] Dark Chemical Matter Library, ChemDiv. https://www.chemdiv.com/catalog/focused-and-targeted-libraries/dark-chemical-matter-library/. Accessed 19 Jan 2026.

[66] Wassermann AM, Lounkine E, Hoepfner D et al (2015) Dark chemical matter as a promising starting point for drug lead discovery. Nat Chem Biol 11:958–966 10.1038/nchembio.1936 26479441

[67] O’Boyle NM, Banck M, James CA et al (2011) Open babel: an open chemical toolbox. J Cheminform 3:33 10.1186/1758-2946-3-33 PMC3198950 21982300

[68] Sievers F, Wilm A, Dineen D et al (2011) Fast, scalable generation of high-quality protein multiple sequence alignments using Clustal Omega. Mol Syst Biol 7:539 10.1038/msb.2011.75 PMC3261699 21988835

[69] Dos Santos RN, Ferreira LG, Andricopulo AD (2018) Practices in molecular docking and structure-based virtual screening. In: Gore M, Jagtap UB (eds) Computational drug discovery and design. Springer, New York, pp 31–50 10.1007/978-1-4939-7756-7_3 29594766

[70] Boltz-2 GitHub page. https://github.com/jwohlwend/boltz/blob/main/docs/prediction.md. Accessed 12 February 2026.

[71] Liu S, Alnammi M, Ericksen SS et al (2019) Practical model selection for prospective virtual screening. J Chem Inf Model 59:282–293 10.1021/acs.jcim.8b00363 PMC6351977 30500183

[72] Fresnais L, Ballester PJ (2021) The impact of compound library size on the performance of scoring functions for structure-based virtual screening. Brief Bioinform 22:bbaa095. 10.1093/bib/bbaa095 32568385

