# Supplementary material for: Enhancing virtual screening of cystathionine β-synthase inhibitors: benchmarking target-specific machine-learning scoring functions against state-of-the-art AI docking and co-folding approaches
Source: J Cheminform. 2026 Jul 18;18:141. doi: 10.1186/s13321-026-01262-x (PMC13670553; doi:10.1186/s13321-026-01262-x)
Supplement: Supplementary file 1 [file 13321_2026_1262_MOESM1_ESM.pdf]

Supporting Information

Enhancing Virtual Screening of Cystathionine β-Synthase Inhibitors: Benchmarking Target-Specific Machine-Learning Scoring Functions Against State-of-the-Art AI Docking and Co-Folding Approaches

Cao-Minh Truong,[1] Van-Thinh To,[1] Nathalie Janel,[2] Julien Dairou,[3] Pedro J. Ballester,[4] Olivier Taboureau,[1] Viet-Khoa Tran-Nguyen[1]*

[1]Université Paris Cité, CNRS UMR 8251, INSERM ERL 1133, F-75013 Paris, France

[2]Université Paris Cité, CNRS UMR 8251, F-75013 Paris, France

[3]Université Paris Cité, CNRS UMR 8601, F-75006 Paris, France

[4]Department of Bioengineering, Imperial College London, London SW7 2AZ, UK

*Corresponding author: Viet-Khoa Tran-Nguyen (viet-khoa.tran-nguyen@u-paris.fr)

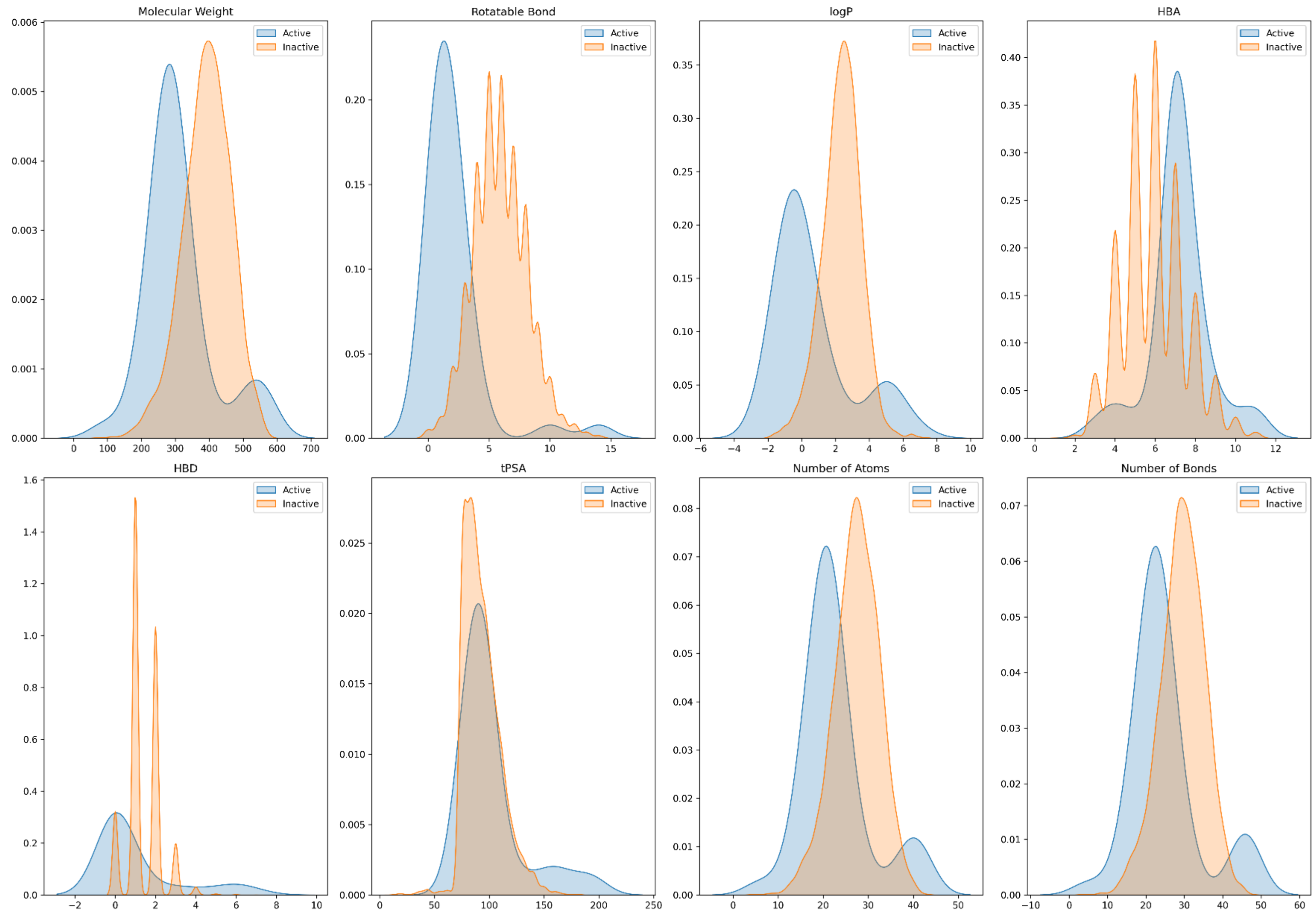

Figure S1. Distributions of eight physicochemical properties for actives and inactives (true inactives and DCM compounds) in the full data set, demonstrating substantial overlap across all properties.

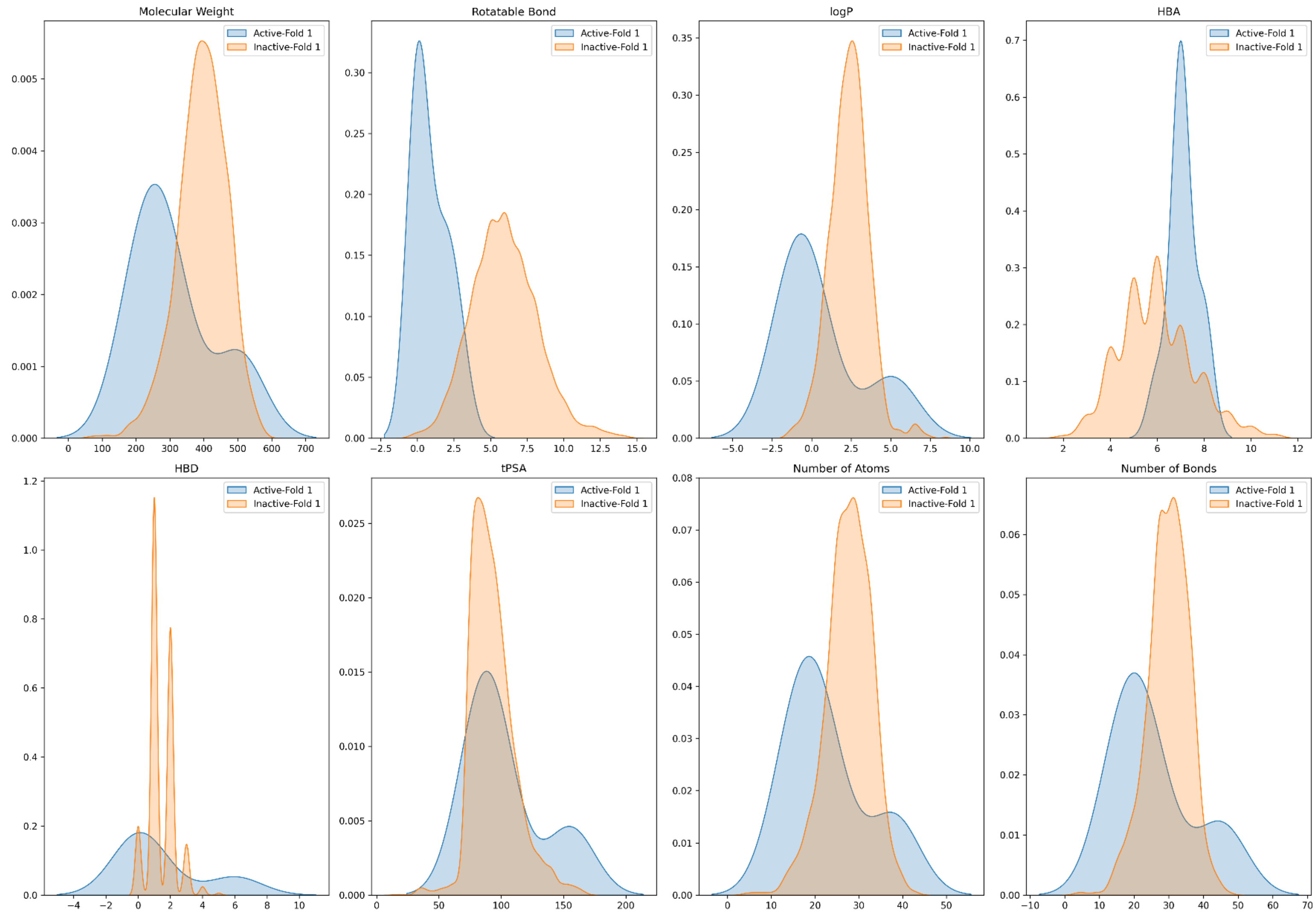

Figure S2. Distributions of eight physicochemical properties for actives and inactives (true inactives and DCM compounds) in fold 1, demonstrating substantial overlap across all properties.

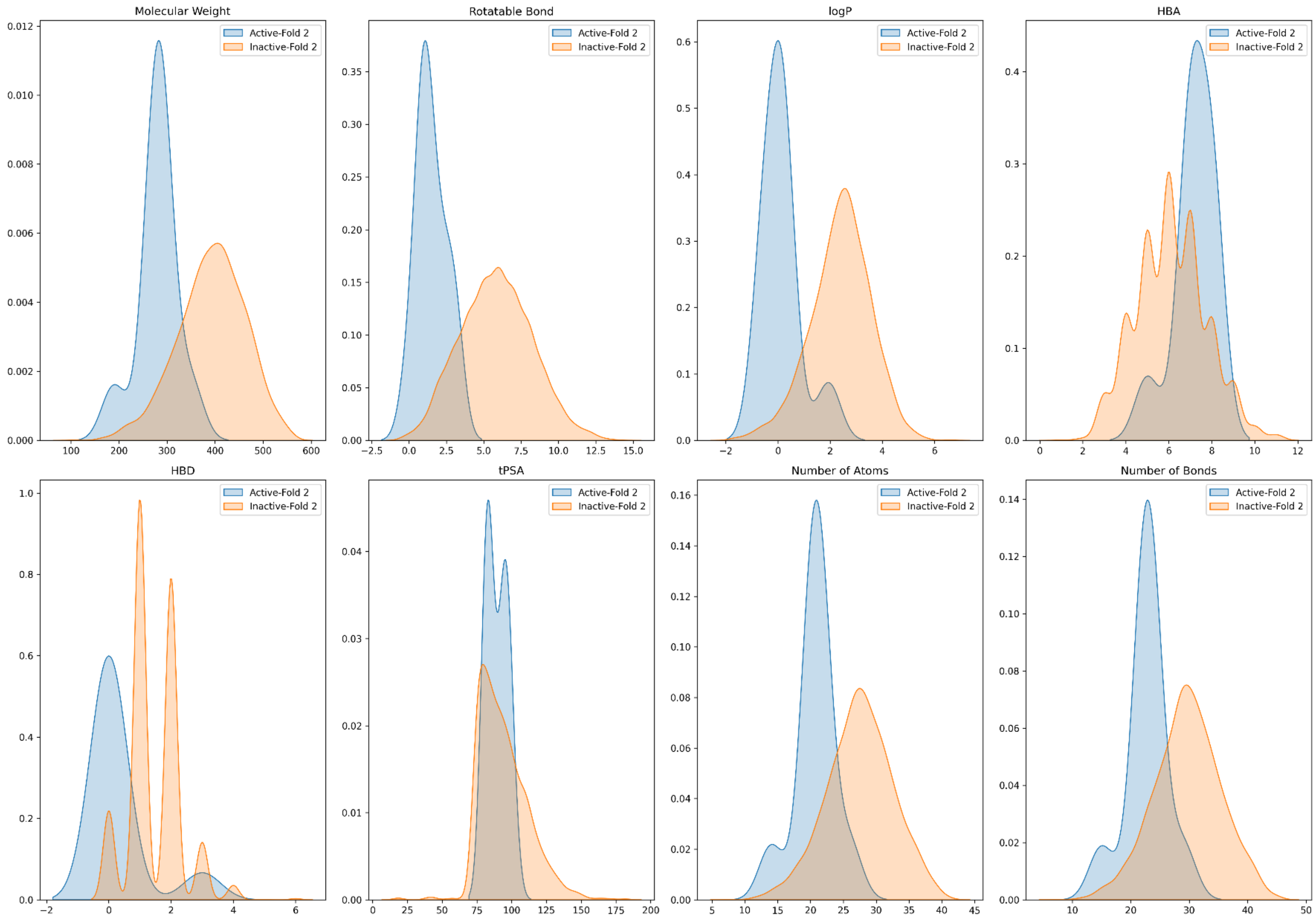

Figure S3. Distributions of eight physicochemical properties for actives and inactives (true inactives and DCM compounds) in fold 2, demonstrating substantial overlap across all properties.

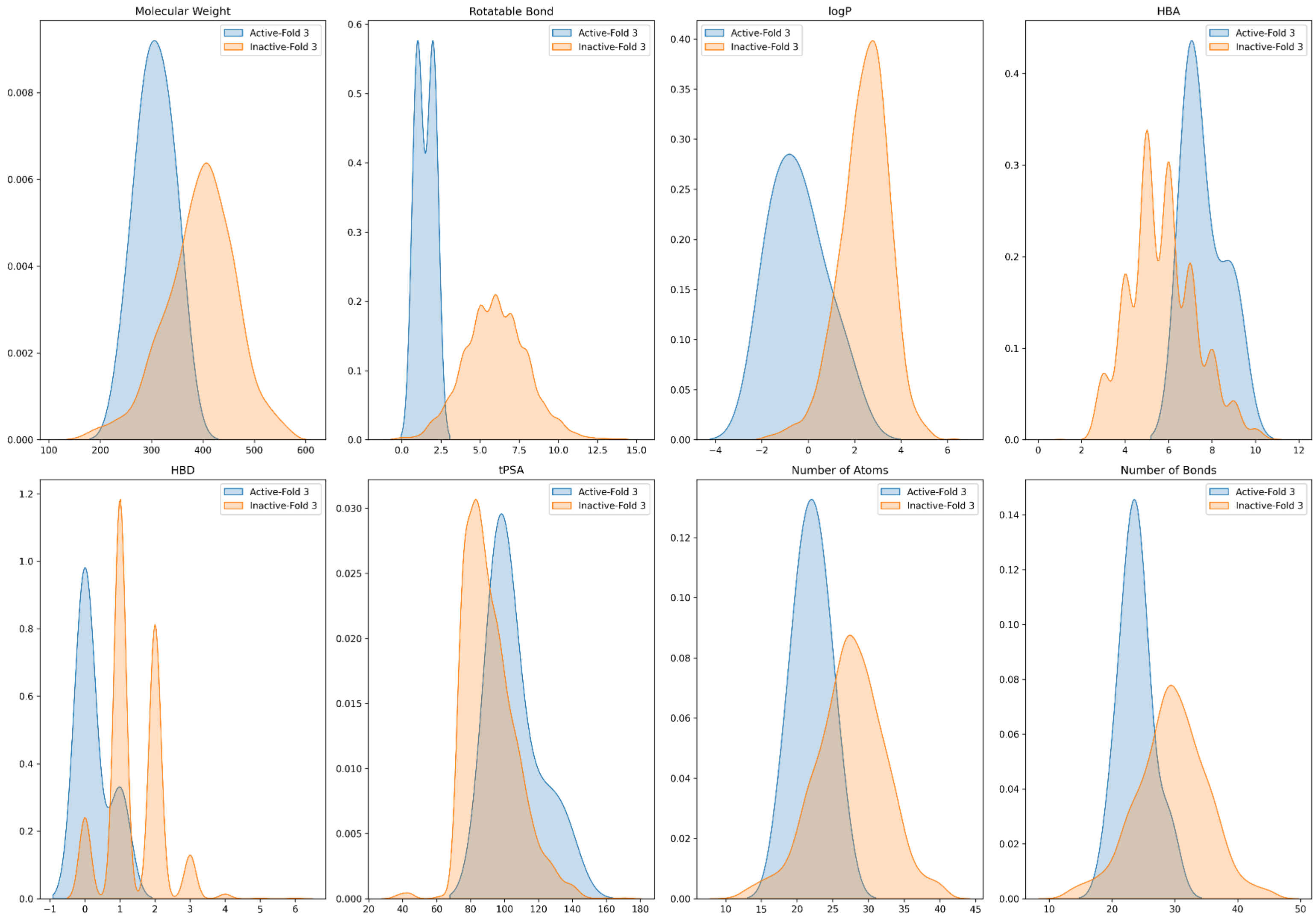

Figure S4. Distributions of eight physicochemical properties for actives and inactives (true inactives and DCM compounds) in fold 3, demonstrating substantial overlap across all properties.

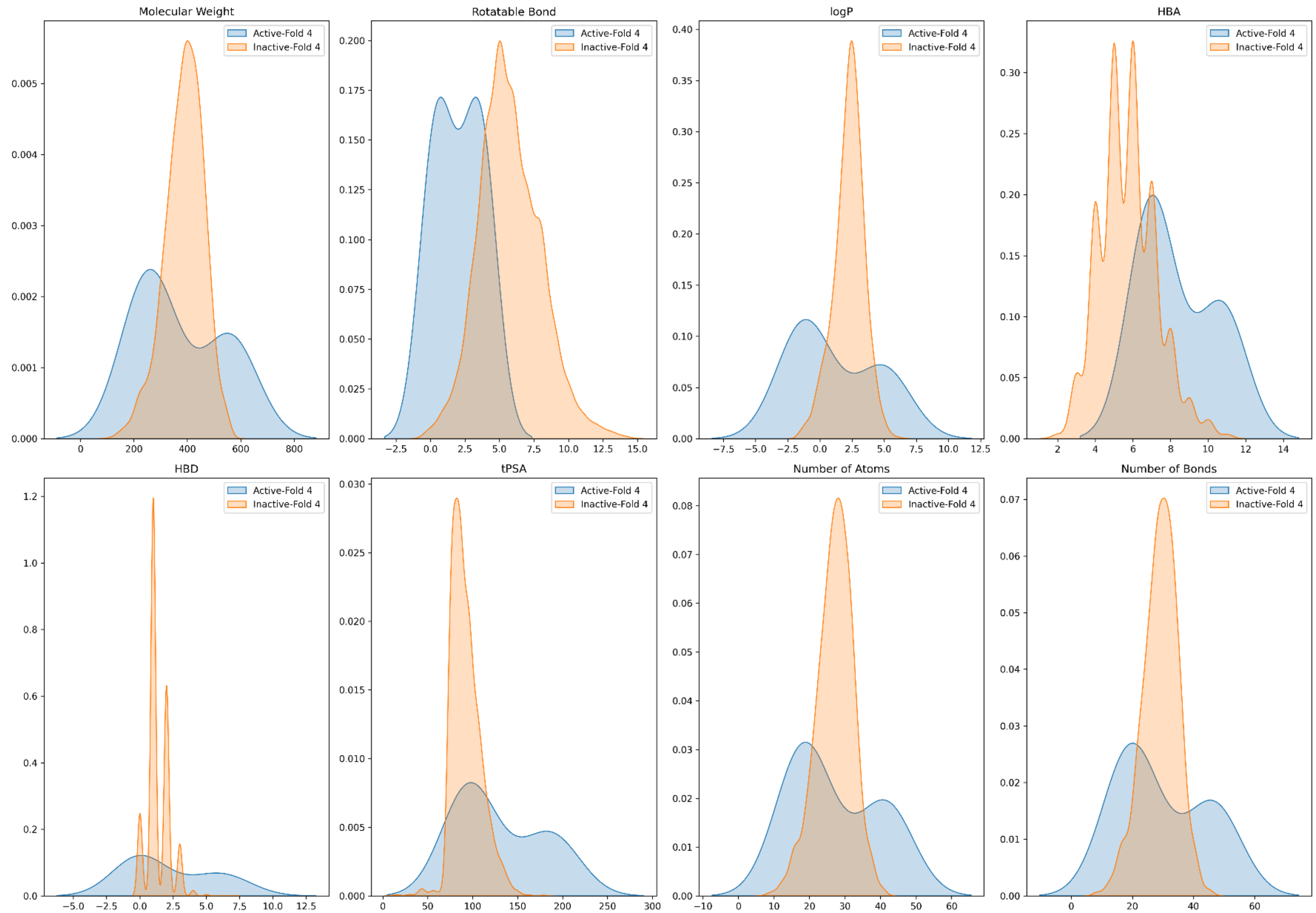

Figure S5. Distributions of eight physicochemical properties for actives and inactives (true inactives and DCM compounds) in fold 4, demonstrating substantial overlap across all properties.

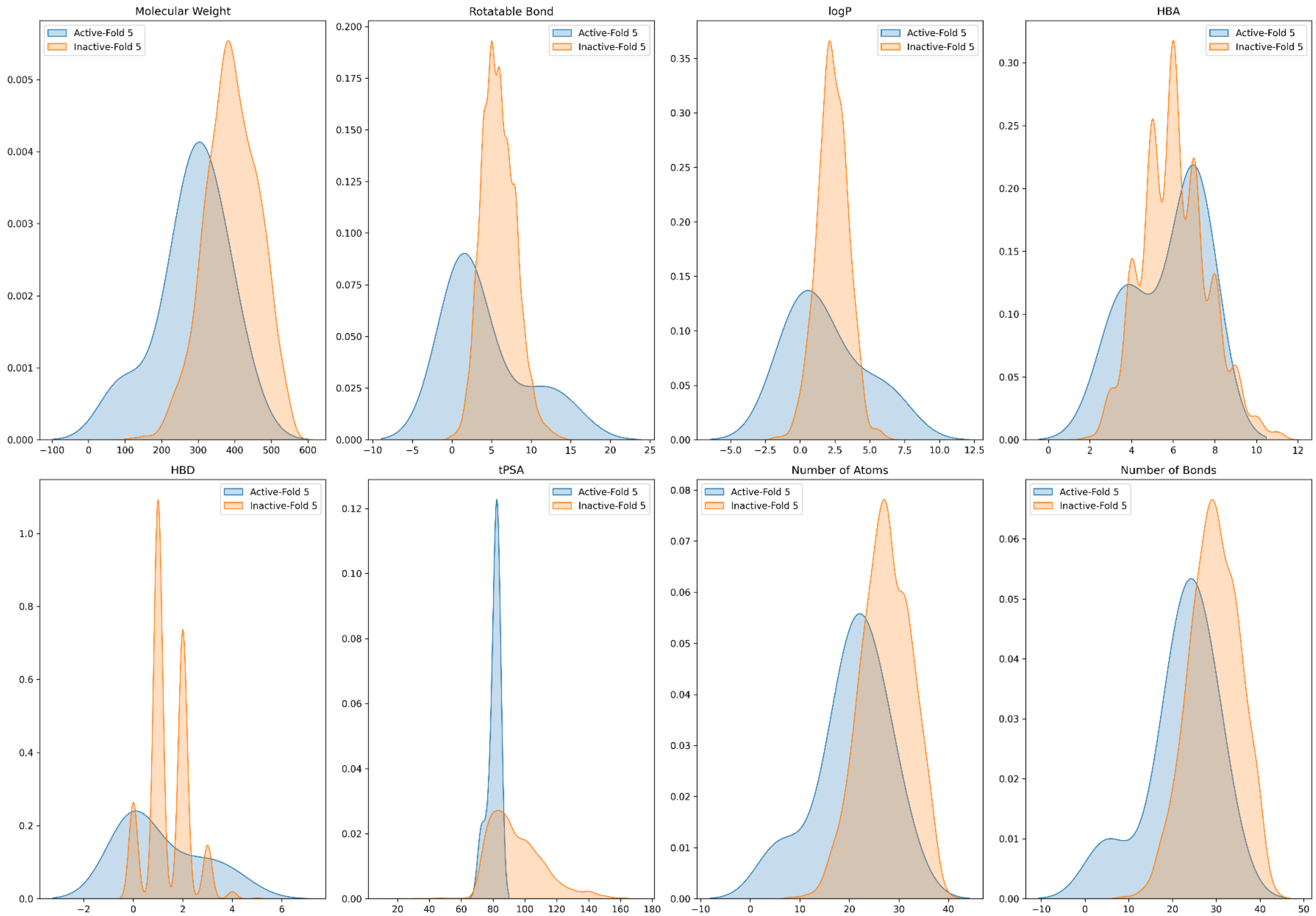

Figure S6. Distributions of eight physicochemical properties for actives and inactives (true inactives and DCM compounds) in fold 5, demonstrating substantial overlap across all properties.

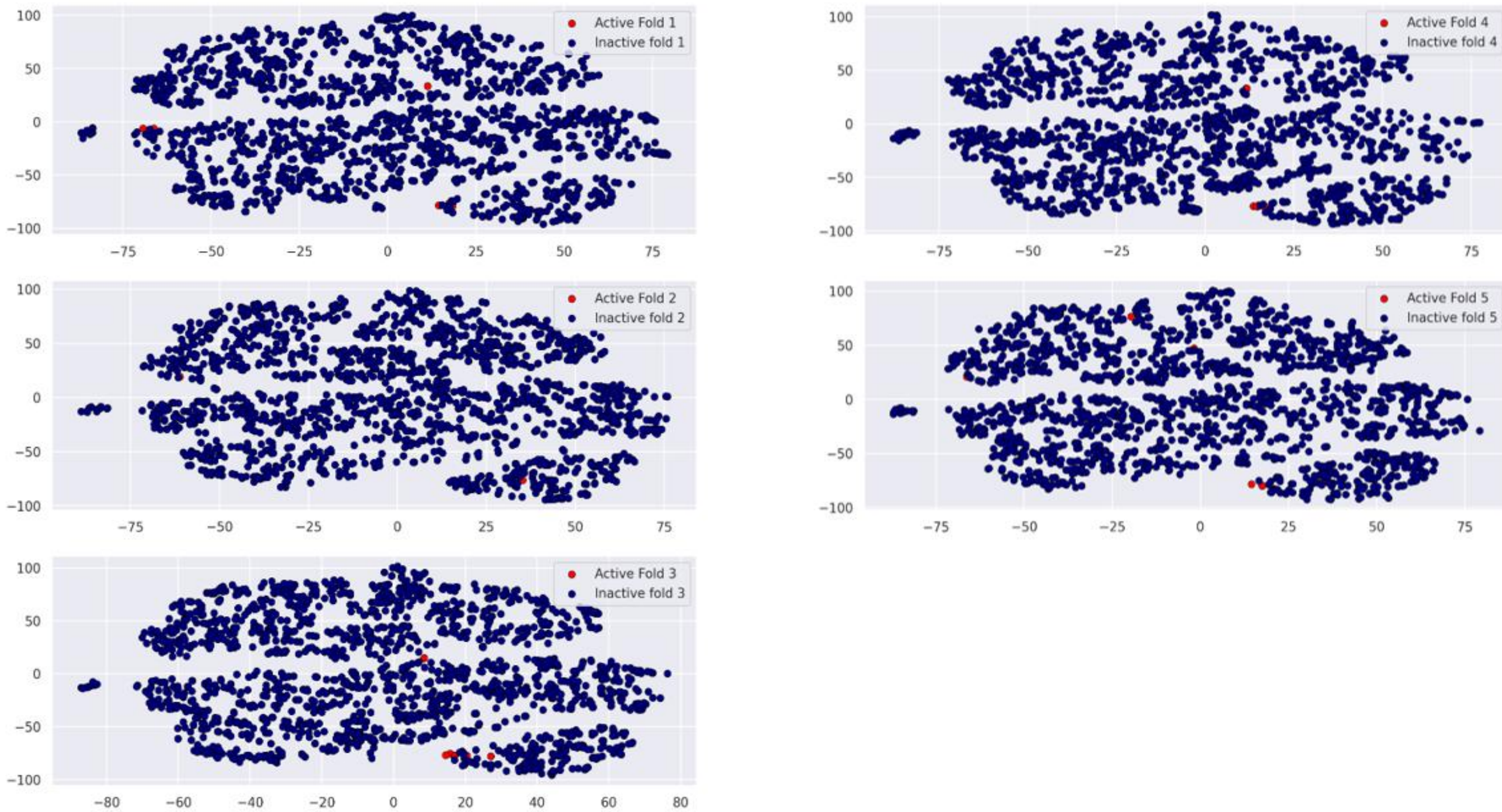

Figure S7. Two-dimensional representation of the chemical space defined by the first two embedded dimensions obtained via t-distributed stochastic neighbor embedding (t-SNE). The analysis was performed on the active and inactive compounds (including both true inactives and DCM compounds) within each of the five folds, using eight physicochemical descriptors (MW, atom and bond counts, logP, hydrogen-bond donor and acceptor counts, rotatable bond count, and TPSA), calculated with RDKit (v.2025.03.2; cf. "Methods") and normalized using the 'StandardScaler' function from 'scikit-learn' (v.1.5.1). Dimensionality reduction was carried out using the 'TSNE' function from 'scikit-learn' (v.1.5.1), with 'n_components' set to 2 and a fixed 'random_state' of 42 (all other parameters set to default). In each fold, active compounds are embedded within the chemical space occupied by inactive compounds, indicating minimal inter-class bias and preservation of the relative chemical space relationship between classes.

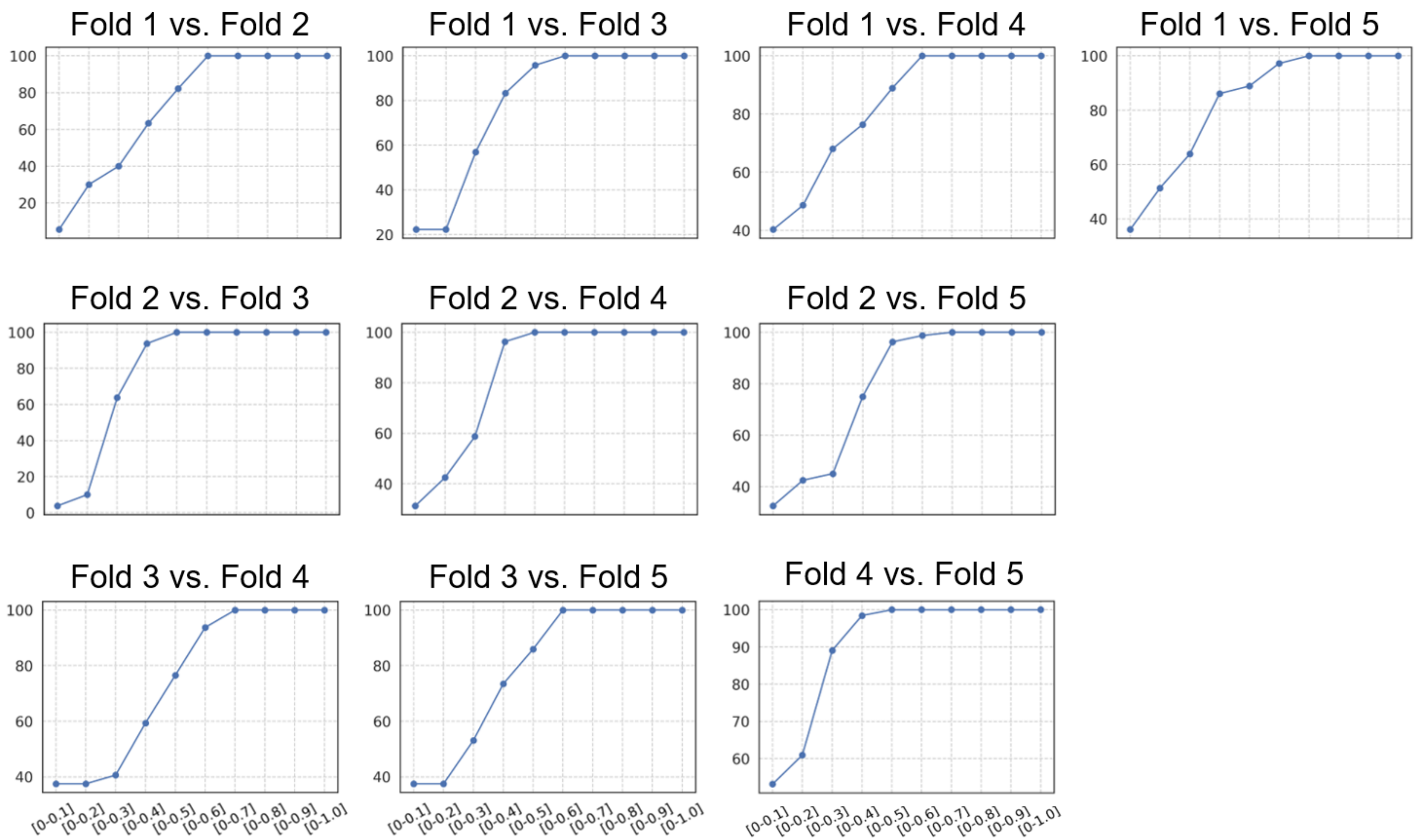

Figure S8. Cumulative percentage of active pairs across the five folds, showing the distribution of Tanimoto similarity coefficients calculated from Morgan fingerprints (2,048 bits, radius 2).

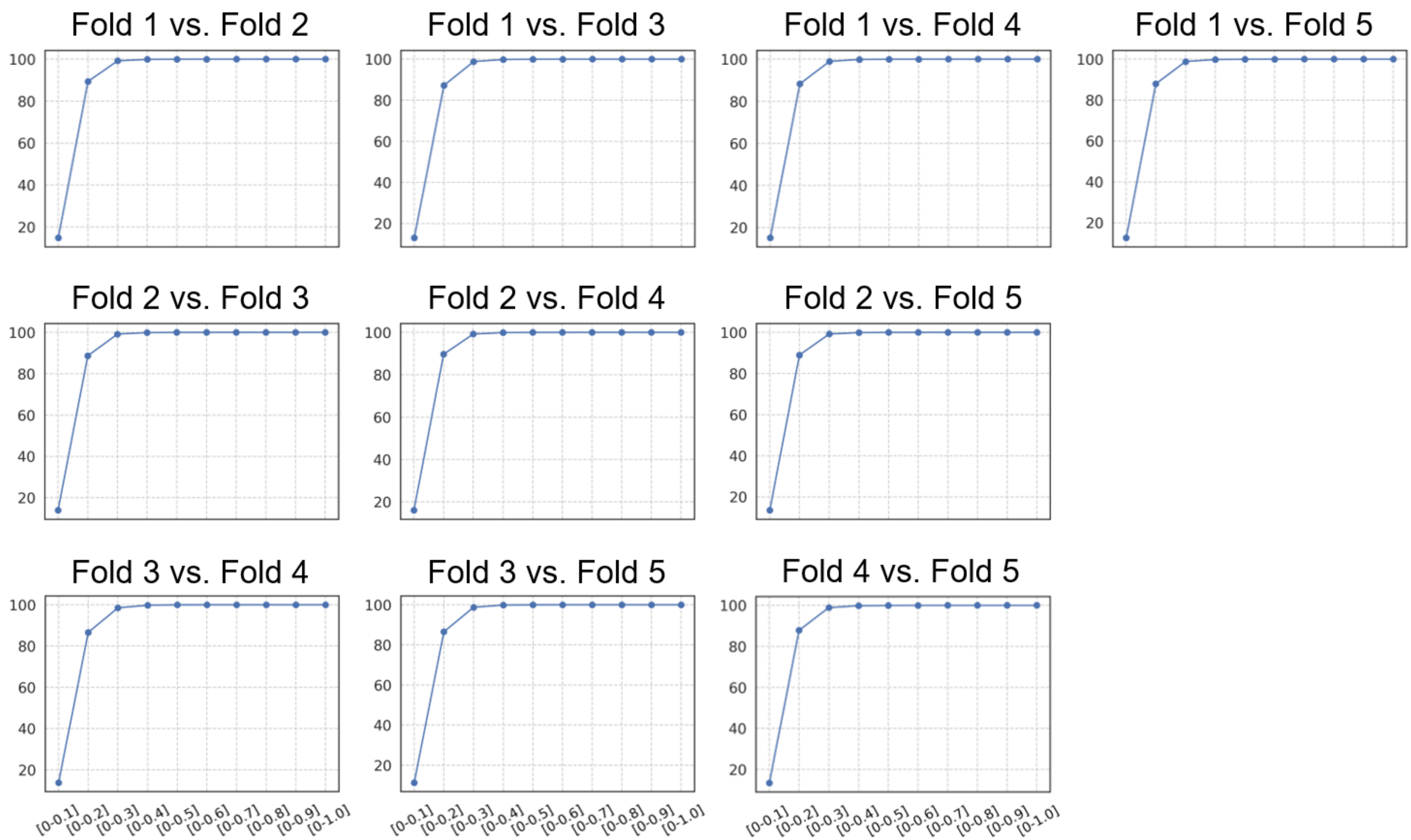

Figure S9. Cumulative percentage of inactive pairs across the five folds, showing the distribution of Tanimoto similarity coefficients calculated from Morgan fingerprints (2,048 bits, radius 2).

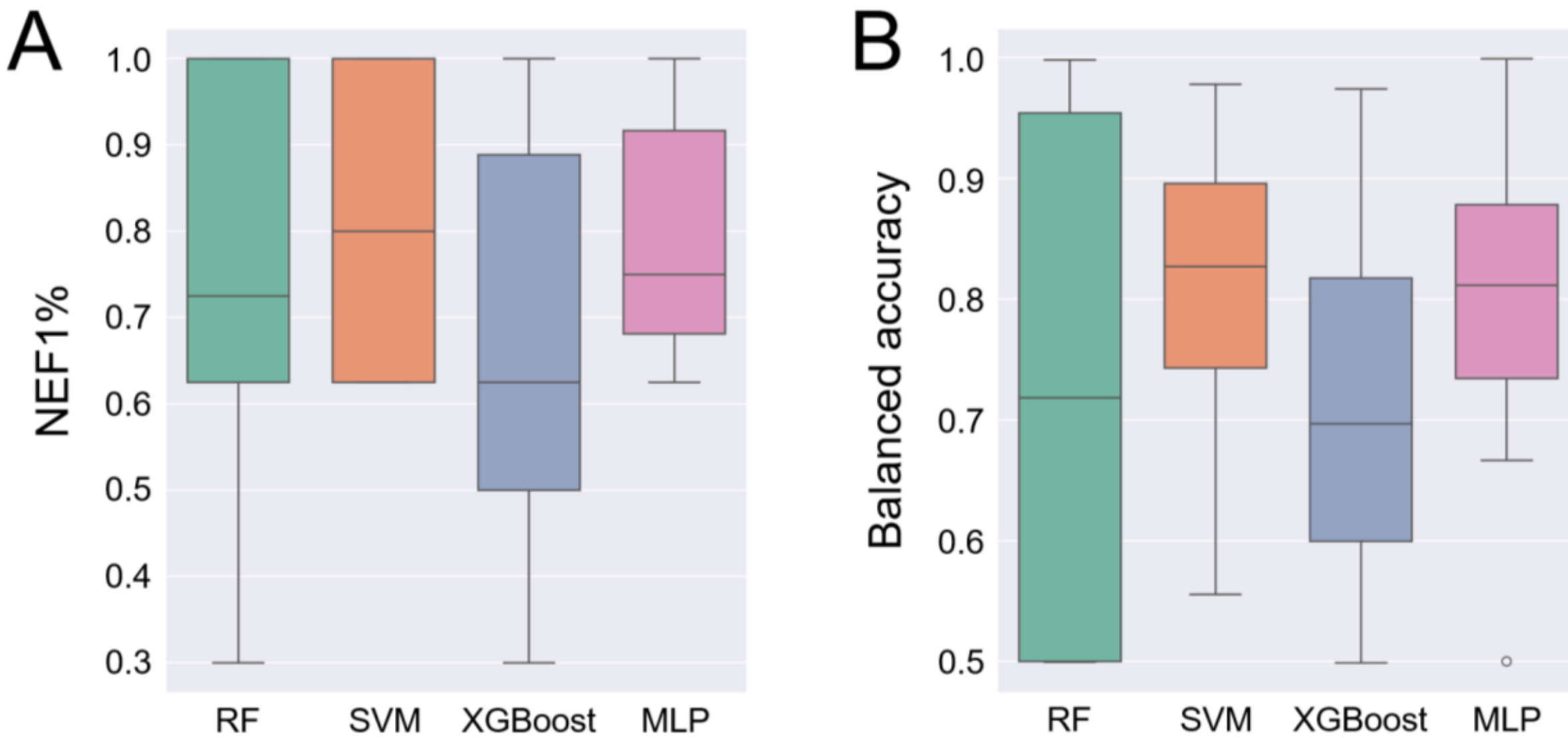

Figure S10. Screening power (A) and balanced accuracy (B) across the five held-out test sets in our CV setup for the four AI algorithms used in our CBS-specific binary classifiers. Neither screening nor classification performance differed significantly among the algorithms (Mann-Whitney U test with Bonferroni correction, p-values > 0.05), indicating comparable robustness across modeling approaches.

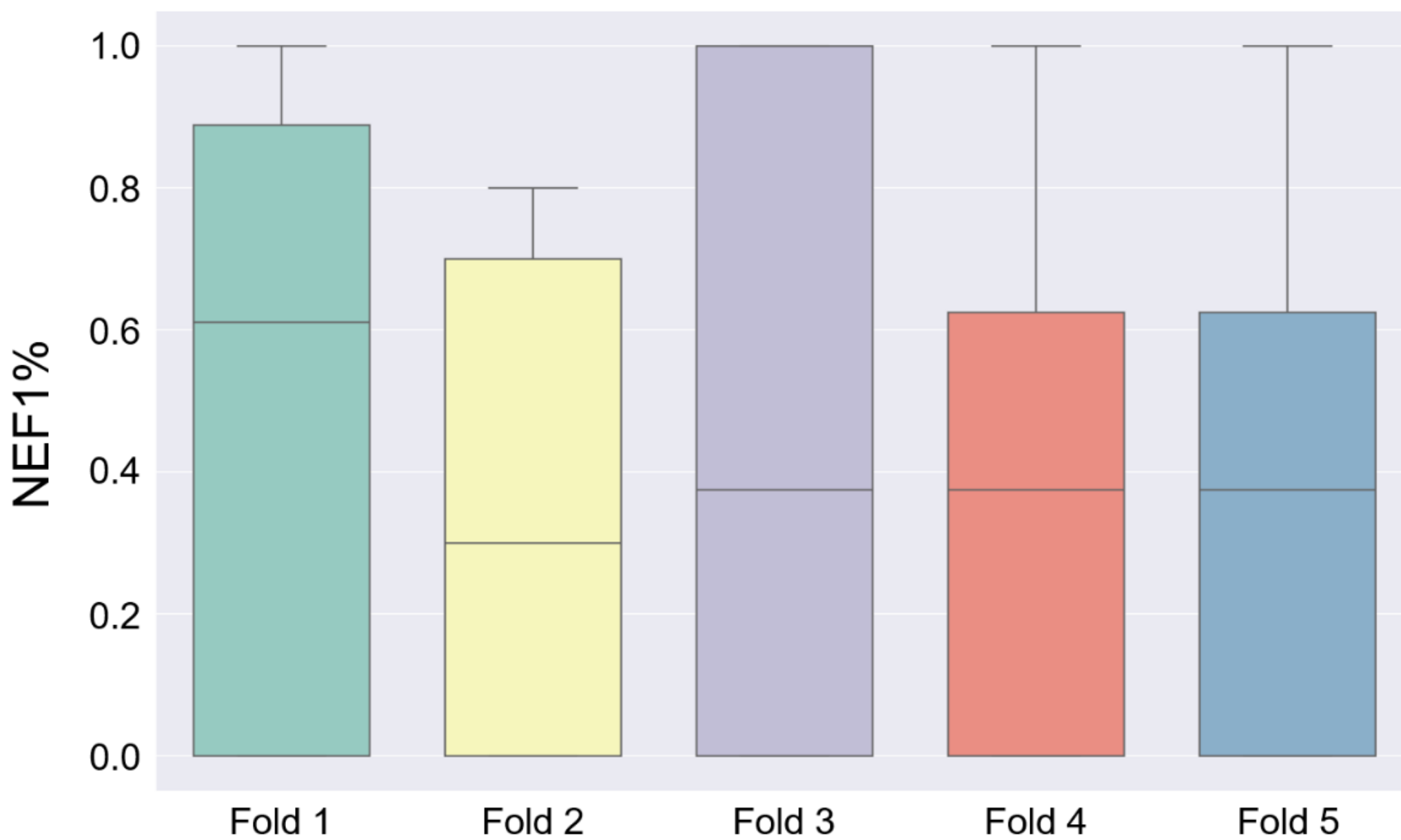

Figure S11. Screening performance (NEF1%) for each fold in our five-fold CV setup. NEF1% values did not differ significantly between folds (Mann-Whitney U test with Bonferroni correction, p-values > 0.05), indicating comparable difficulty across the test partitions. This result confirms that the UMAP-based clustering combined with similarity-controlled splitting generated structurally independent folds with balanced challenge levels, supporting a fair comparison between the CBS-specific models and the reference VS pipelines.

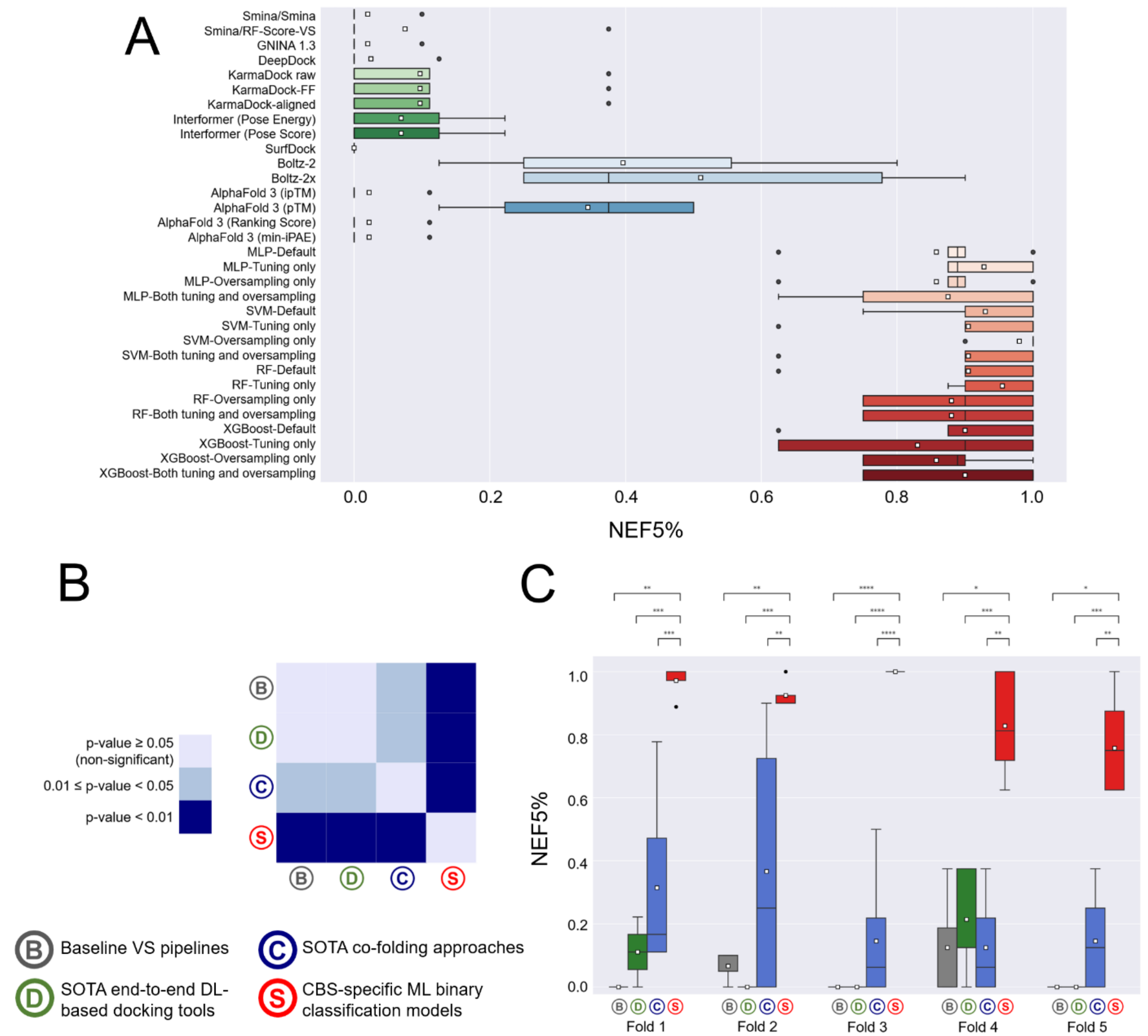

Figure S12. Screening power across the five folds of our CV setup for 16 existing structure-based VS pipelines and our CBS-specific AI binary classifiers based on four algorithms (RF, XGBoost, SVM, and MLP). (A) Box-and-whisker plots showing the NEF5% distribution across the five held-out test sets for all 32 evaluated VS pipelines (16 generic pipelines and 16 CBS-specific models). (B) Heatmap of pairwise p-values comparing screening power (NEF5%) between pipeline categories (baseline VS pipelines, SOTA end-to-end DL-based docking tools, SOTA co-folding approaches, and CBS-specific AI classifiers) across the five test folds. (C) Box-and-whisker plots showing per-fold screening power (NEF5%) for each VS pipeline category. For panels (A) and (C), white squares indicate mean values, the horizontal line inside each box represents the median, and filled circles

denote outliers defined using the 1.5IQR rule. For panels (B) and (C), statistical comparisons were performed using the Mann-Whitney U test with Bonferroni correction, implemented via the 'Annotator' function from the 'statannotations.Annotator' module ('statannotations' Python package, v.0.7.2). In (C), only statistically significant comparisons are displayed, with significance levels indicated as follows: * ($0.01 < p\text{-value} \leq 0.05$), ** ($0.001 < p\text{-value} \leq 0.01$), *** ($0.0001 < p\text{-value} \leq 0.001$), and **** ($p\text{-value} < 0.0001$). Panels (B) and (C) together show that CBS-specific AI pipelines consistently outperformed the 16 existing VS pipelines both overall and on individual folds.

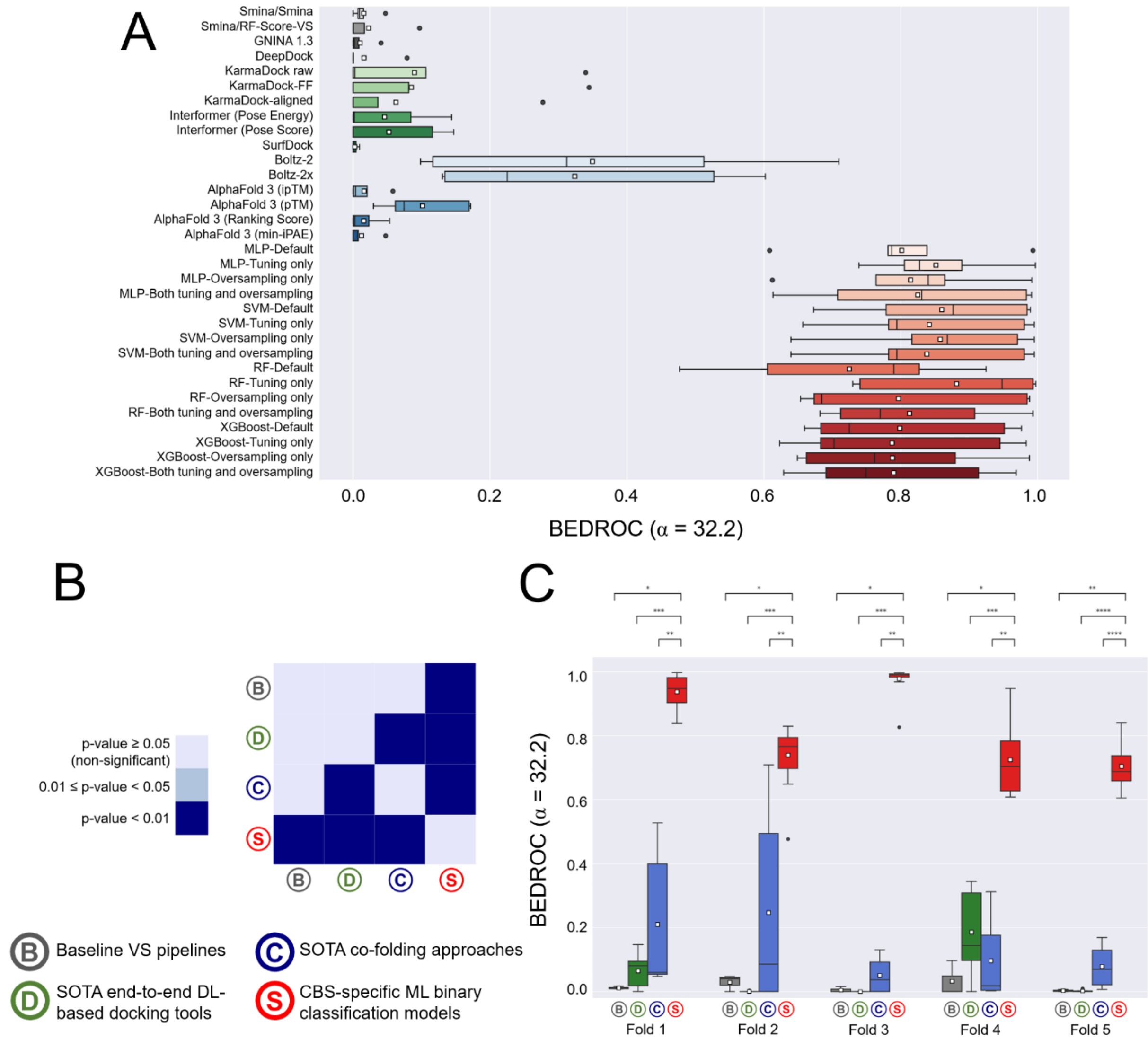

Figure S13. Screening power across the five folds of our CV setup for 16 existing structure-based VS pipelines and our CBS-specific AI binary classifiers based on four algorithms (RF, XGBoost, SVM, and MLP). (A) Box-and-whisker plots showing the BEDROC score (α = 32.2: 80% of the score arising from the top 5% of the ranked list) distribution across the five held-out test sets for all 32 evaluated VS pipelines (16 generic pipelines and 16 CBS-specific models). (B) Heatmap of pairwise p-values comparing screening power (BEDROC score, α = 32.2) between pipeline categories (baseline VS pipelines, SOTA end-to-end DL-based docking tools, SOTA co-folding approaches, and CBS-specific AI classifiers) across the five test folds. (C) Box-and-whisker plots showing per-fold screening power (BEDROC score, α = 32.2) for each VS pipeline category. For panels (A) and (C), white squares indicate mean values, the horizontal line inside each box represents the median,

and filled circles denote outliers defined using the 1.5IQR rule. For panels (B) and (C), statistical comparisons were performed using the Mann-Whitney U test with Bonferroni correction, implemented via the ‘Annotator’ function from the ‘statannotations.Annotator’ module (‘statannotations’ Python package, v.0.7.2). In (C), only statistically significant comparisons are displayed, with significance levels indicated as follows: * (0.01 < p-value ≤ 0.05), ** (0.001 < p-value ≤ 0.01), *** (0.0001 < p-value ≤ 0.001), and **** (p-value < 0.0001). Panels (B) and (C) together show that CBS-specific AI pipelines consistently outperformed the 16 existing VS pipelines both overall and on individual folds.

Table S1. Details of UMAP-based clusters for active and inactive compounds in the final data set.

	Active clusters	Inactive clusters
Number of clusters (‘n_clusters’)#	11	2,570
‘n_neighbors’#	2	2
‘min_dist’#	0.12321567138160146	0.005633951252083743
Silhouette score	0.94	0.97
Calinski-Harabasz index	3656.1826	3076791.1361
Davies-Bouldin index	0.0686	0.0507

#These hyperparameters were tuned using Optuna (v.4.4.0) over 100 optimization trials with a Tree-structured Parzen Estimator (TPE) sampler to optimize the clustering procedure. The silhouette score was used as the optimization objective. This hyperparameter optimization was conducted independently for the actives and the inactives.

Table S2. Screening performance (NEF1%) for each fold of our five-fold CV setup for the 16 existing VS pipelines and the CBS-specific AI binary classification models.

VS pipelines	Fold 1	Fold 2	Fold 3	Fold 4	Fold 5
Baseline VS pipelines					
Smina scoring of Smina docking poses	0.000	0.000	0.000	0.000	0.000
RF-Score-VS rescoring of Smina poses	0.000	0.000	0.000	0.000	0.000
GNINA 1.3 docking with CNN-based scoring	0.000	0.000	0.000	0.000	0.000
SOTA end-to-end DL-based docking tools					
DeepDock	0.000	0.000	0.000	0.000	0.000
KarmaDock raw	0.111	0.000	0.000	0.250	0.000
KarmaDock-FF	0.000	0.000	0.000	0.250	0.000
KarmaDock-aligned	0.000	0.000	0.000	0.250	0.000
Interformer (Pose Energy)	0.000	0.000	0.000	0.125	0.000
Interformer (Pose Score)	0.111	0.000	0.000	0.125	0.000
SurfDock	0.000	0.000	0.000	0.000	0.000
SOTA co-folding approaches					
Boltz-2	0.444	0.600	0.000	0.250	0.000
Boltz-2x	0.333	0.400	0.000	0.125	0.000
AlphaFold 3 (ipTM)	0.000	0.000	0.000	0.000	0.000
AlphaFold 3 (pTM)	0.000	0.200	0.000	0.000	0.125
AlphaFold 3 (Ranking Score)	0.000	0.000	0.000	0.000	0.000
AlphaFold 3 (min-iPAE)	0.000	0.000	0.000	0.000	0.000
Our CBS-specific AI binary classification models					
RF-Default	0.889	0.300	0.750	0.750	0.625
RF-Tuning only	1.000	0.600	1.000	1.000	0.625
RF-Oversampling only	1.000	0.600	1.000	0.625	0.625
RF-Both tuning and oversampling	0.778	0.700	1.000	0.625	0.625
SVM-Default	1.000	0.700	1.000	0.625	0.625

SVM-Tuning only	1.000	0.800	1.000	0.625	0.625
SVM-Oversampling only	1.000	0.800	1.000	0.625	1.000
SVM-Both tuning and oversampling	1.000	0.800	1.000	0.625	0.625
XGBoost-Default	0.889	0.300	1.000	0.625	0.625
XGBoost-Tuning only	0.889	0.500	1.000	0.625	0.625
XGBoost-Oversampling only	0.889	0.300	1.000	0.500	0.750
XGBoost-Both tuning and oversampling	0.778	0.500	1.000	0.500	0.625
MLP-Default	0.778	0.700	1.000	0.625	0.750
MLP-Tuning only	0.889	0.700	1.000	0.625	0.750
MLP-Oversampling only	0.778	0.700	1.000	0.625	0.750
MLP-Both tuning and oversampling	1.000	0.700	1.000	0.625	0.625

Table S3. Classification performance (balanced accuracy) for each fold of our five-fold CV setup for the CBS-specific AI binary classification models.

AI binary classification models	Fold 1	Fold 2	Fold 3	Fold 4	Fold 5
RF-Default	0.5000	0.5000	0.5000	0.5000	0.5000
RF-Tuning only	0.9896	0.9372	0.9983	0.9964	0.8120
RF-Oversampling only	0.5000	0.4995	0.5625	0.6250	0.5625
RF-Both tuning and oversampling	0.9348	0.9572	0.9728	0.9532	0.8582
SVM-Default	0.7222	0.8464	0.8745	0.6870	0.7500
SVM-Tuning only	0.9428	0.8957	0.9779	0.8673	0.8425
SVM-Oversampling only	0.5556	0.5990	0.7500	0.6875	0.7500
SVM-Both tuning and oversampling	0.9425	0.8962	0.9373	0.8094	0.8113
XGBoost-Default	0.6111	0.4988	0.7500	0.6248	0.5625
XGBoost-Tuning only	0.7068	0.8328	0.5649	0.6481	0.5000
XGBoost-Oversampling only	0.6667	0.5478	0.8750	0.6873	0.8125
XGBoost-Both tuning and oversampling	0.9742	0.9351	0.9351	0.7459	0.8106
MLP-Default	0.6667	0.7978	0.8748	0.6868	0.6875
MLP-Tuning only	0.8886	0.8959	0.5000	0.8106	0.7500
MLP-Oversampling only	0.7778	0.8471	0.8748	0.6865	0.8125
MLP-Both tuning and oversampling	0.9432	0.8962	0.9990	0.8082	0.8125

Table S4. Hyperparameter search space for the CBS-specific binary classifiers, optimized by maximizing the average NEF1% from four-fold internal CV on the training data of each split in our five-fold CV setup.

Algorithm	Hyperparameters	Search space
RF	n_estimators	[100, 1000]
	max_depth	[2, 16]
	min_samples_split	[2, 10]
	min_samples_leaf	[1, 10]
	max_features	[‘sqrt’, ‘log2’]
	criterion	[‘gini’, ‘entropy’, ‘log_loss’]
SVM	C	[1e-1, 10] (log=True)
	kernel	[‘rbf’, ‘linear’]
	gamma	[1e-2, 1] (log=True)
XGBoost	n_estimators	[100, 1000]
	max_depth	[2, 10]
	learning_rate	[1e-3, 0.3] (log=True)
	subsample	[0.1, 1]
	colsample_bytree	[0.1, 1]
	gamma	[1e-4, 10] (log=True)
	min_child_weight	[0, 10]
	reg_lambda	[1e-3, 10] (log=True)
	reg_alpha	[1e-3, 10] (log=True)
MLP	layer1_nodes	[10, 1000]
	layer2_nodes	[10, 1000]
	layer3_nodes	[10, 1000]
	hidden_layer_sizes	(layer1_nodes, layer2_nodes, layer3_nodes)
	activation	[‘relu’, ‘tanh’, ‘logistic’]

	solver	['adam', 'sgd']
	alpha	[1e-5, 1e-1] (log=True)
	batch_size	[8, 1024]
	learning_rate_init	[1e-4, 1e-1] (log=True)
	max_iter	200

Table S5. Optimal hyperparameters and decision thresholds for the tuned CBS-specific AI classification models, obtained from the training portion of each split in our five-fold CV setup.

Training data	Folds 2-5	Folds 1, 3-5	Folds 1, 2, 4, 5	Folds 1-3, 5	Folds 1-4
RF-Tuning only					
n_estimators	367	847	271	959	792
max_depth	9	9	9	12	13
min_samples_split	9	3	3	4	3
min_samples_leaf	2	1	5	1	1
max_features	log2	log2	sqrt	log2	log2
criterion	entropy	log_loss	entropy	log_loss	log_loss
Decision threshold	0.013724345130263601	0.017796498573580143	0.02322076031346345	0.020530209687031807	0.023055968596733548
RF-Both tuning and oversampling					
n_estimators	711	1000	877	623	524
max_depth	15	16	16	16	15
min_samples_split	9	9	10	8	10
min_samples_leaf	3	5	6	10	8
max_features	log2	sqrt	sqrt	log2	log2
criterion	entropy	gini	entropy	entropy	entropy
Decision threshold	0.030195884398005145	0.015908649674522962	0.020530209687031807	0.029093390831862524	0.03048300876715799
SVM-Tuning only					
C	1.6738085788752133	1.6738085788752133	1.6738085788752133	1.6738085788752133	1.6738085788752133
kernel	linear	linear	linear	linear	linear
gamma	0.05404103854647329	0.05404103854647329	0.05404103854647329	0.05404103854647329	0.05404103854647329
Decision threshold	0.05078882905494822	0.09304392500886602	0.004603261777884557	0.013383880430635051	0.003301081858218765
SVM-Both tuning and oversampling					
C	1.6738085788752133	1.6738085788752133	1.6738085788752133	1.6738085788752133	1.6738085788752133
kernel	linear	linear	linear	linear	linear
gamma	0.05404103854647329	0.05404103854647329	0.05404103854647329	0.05404103854647329	0.05404103854647329
Decision threshold	0.0002066617507246151	0.027826845382009628	0.00020729100724893525	0.00020725824825967311	7.690632775938415e-5

XGBoost-Tuning only					
n_estimators	572	335	686	714	972
max_depth	8	2	4	6	5
learning_rate	0.09264138549862595	0.1481132643760312	0.04093593258977919	0.038518678192541955	0.013258738613900579
subsample	0.5551788878114882	0.36571208061136623	0.15128134826945755	0.5303304236400109	0.5161518139904514
colsample_bytree	0.5740428802308275	0.2224838200647238	0.2313795760465189	0.37428233723633564	0.10074175774444133
gamma	0.3833741897272174	0.00016966291662035886	0.004767000707956224	0.0025065815043713	2.626487127562966
min_child_weight	4.996337325817175	0.11015267411122898	0.5070249169738635	4.4492237694713435	0.2832609617644253
reg_lambda	4.951309947486161	0.13965772111634814	0.11883575505352234	5.014447788887427	0.0541706699416274
reg_alpha	1.0088373918499682	0.03075760689517225	0.18254707478717708	0.0089878485651255	0.019089987473042514
Decision threshold	0.03542451910217835	0.001389191590628122	0.001281693641841608	0.02424635928743875	0.0018490064927650998
XGBoost-Both tuning and oversampling					
n_estimators	928	965	140	482	737
max_depth	2	5	2	5	2
learning_rate	0.17852946374745765	0.03409707261754395	0.16555385678193887	0.029989179105368243	0.2526878207508456
subsample	0.7792344898533413	0.5601305501717242	0.726026310798668	0.10883137832358161	0.8491983767203796
colsample_bytree	0.20222354703750003	0.3085532444016241	0.9849147024308079	0.446943137765372	0.29110519961044856
gamma	0.0003054285799668561	0.0024990985190614093	0.0003753245061467077	0.7309056858786138	0.0008111941985431928
min_child_weight	1.3602375313958737	2.5391071371814937	2.383326454286155	2.9166642509274605	1.8340450985343382
reg_lambda	0.001341358275148361	0.004549379633110634	0.07294011215423794	0.6691455190994686	0.016480446427978974
reg_alpha	0.22361885014389	0.6719418872918791	0.09901969064726132	0.0024448465209117977	0.12561043700013558
Decision threshold	0.00020666178230992086	0.0036727492392132494	0.021385010366970506	0.0834661698416595	0.016829860423956226
MLP-Tuning only					
hidden_layer_sizes	(86, 419, 838)	(878, 64, 930)	(710, 300, 989)	(543, 898, 557)	(664, 883, 374)
activation	relu	logistic	tanh	tanh	logistic
solver	adam	adam	adam	adam	adam
alpha	2.3646272727489918e-05	0.00037230950665203194	5.8890553972566604e-05	0.00014270379857934224	0.0003833083258575713
batch_size	551	91	429	222	537
learning_rate_init	0.003746097171179446	0.00412557907693281	0.0030171288204151572	0.0013586849561974376	0.0012752912235425291
Decision threshold	0.00039048571093086744	0.00976306226786305	0.00019038590962325362	7.798671574328793e-05	0.3745401188473625

MLP-Both tuning and oversampling					
hidden_layer_sizes	(706, 152, 173)	(339, 718, 275)	(801, 341, 67)	(181, 663, 67)	(266, 666, 318)
activation	tanh	tanh	tanh	logistic	tanh
solver	adam	adam	adam	adam	adam
alpha	4.455981748912941e-05	0.02064977618526575	0.0031860882600830585	0.0016576415806465127	0.057279044707996205
batch_size	742	962	751	407	918
learning_rate_init	0.0044729366236088575	0.001990803158675304	0.007376856672217021	0.00022390177981576696	0.00621870472776908
Decision threshold	0.00020625845628798089	0.00018969750940739535	0.0003906085282907425	0.0018490064927650998	0.003344368188415197

Data S1. Supporting data for additional analyses of the data set and VS performance.

S1.1. Ligand-derived RDKit descriptor-based logistic regression models.

A total of 217 ligand-based physicochemical descriptors were computed using the 'Descriptors.CalcMolDescriptors' function from RDKit (v.2025.03.2). The resulting feature matrix was scaled using the 'preprocessing.Normalizer' function from 'scikit-learn' (v.1.5.1), fitted on the training folds and applied to the corresponding test fold within our five-fold CV framework. For each of the five training-test splits, a logistic regression model was trained using these normalized descriptors and the 'LogisticRegression' function from 'scikit-learn' (v.1.5.1) with default hyperparameters.

The full feature matrix containing the 217 RDKit descriptors for all molecules in the data set, as well as the held-out-fold predictions of each logistic regression model, are available in our GitHub repository (https://github.com/caominhtr/CBS/tree/main/data/physchem_property).

The screening power (NEF1%) of these ligand-based logistic regression models on their corresponding held-out test fold was as follows:

Fold 1 = 0.111, Fold 2 = 0.100, Fold 3 = 0.000, Fold 4 = 0.000, and Fold 5 = 0.125.

Fold-wise comparisons of NEF1% values between the ligand-based logistic regression models and the corresponding CBS-specific AI approaches were performed using a one-sample Wilcoxon signed-rank test with Bonferroni correction (five comparisons). The adjusted p-values were as follows:

Fold 1 = 0.0018, Fold 2 = 0.0020, Fold 3 = 0.0005, Fold 4 = 0.0011, and Fold 5 = 0.0013.

All adjusted p-values were below 0.05, indicating statistically significant differences in all folds. These results suggest that, within each fold, models trained on the remaining training data were unable to effectively exploit ligand-derived physicochemical descriptors for prioritizing true CBS inhibitors. In contrast, our CBS-specific structure-based AI approaches consistently outperformed these ligand-based models, in terms of screening power, across all folds.

S1.2. Concatenated evaluation setting.

A concatenated evaluation setting was also considered. In this setting, held-out-fold predictions from all five folds were concatenated for each of our 16 CBS-specific AI approaches, as well as for the 16 existing screening pipelines, and the NEF1% was subsequently computed on the resulting complete data set. All experimentally confirmed actives were thus considered simultaneously in the evaluation. The NEF1% values of the 16 CBS-specific AI approaches and the 16 existing generic pipelines in this concatenated evaluation are reported in the following table.

16 existing generic VS pipelines		16 CBS-specific AI approaches	
Smina/Smina	0.000	RF-Default	0.651
Smina/RF-Score-VS	0.000	RF-Oversampling only	0.791
GNINA 1.3	0.000	RF-Tuning only	0.884
DeepDock	0.000	RF-Both tuning and oversampling	0.767
KarmaDock raw	0.070	SVM-Default	0.837
KarmaDock-FF	0.047	SVM-Oversampling only	0.767
KarmaDock-aligned	0.047	SVM-Tuning only	0.837
Interformer (Pose Energy)	0.023	SVM-Both tuning and oversampling	0.791
Interformer (Pose Score)	0.047	MLP-Default	0.767
SurfDock	0.000	MLP-Oversampling only	0.791
Boltz-2	0.302	MLP-Tuning only	0.698
Boltz-2x	0.186	MLP-Both tuning and oversampling	0.744
AlphaFold 3 (ipTM)	0.000	XGBoost-Default	0.744
AlphaFold 3 (pTM)	0.023	XGBoost-Oversampling only	0.744
AlphaFold 3 (Ranking Score)	0.000	XGBoost-Tuning only	0.698
AlphaFold 3 (min-iPAE)	0.000	XGBoost-Both tuning and oversampling	0.721
Mean NEF1%: 0.047		Mean NEF1%: 0.765	

Comparison of the NEF1% values of the 16 CBS-specific AI approaches and the 16 existing generic VS pipelines in this concatenated evaluation yielded a statistically significant difference (Mann-Whitney U test with Bonferroni correction, p-value = 1×10^{-6}). This result demonstrates that our CBS-specific AI approaches significantly outperformed the 16 existing pipelines, in terms of screening power, in this concatenated setting.

Comparison between the concatenated-setting screening performance of all evaluated approaches and their corresponding fold-wise screening performance in terms of NEF1% gave the following results:

Concatenated evaluation: NEF1% = 0.406 ± 0.366;

Fold-wise evaluation: NEF1% = 0.405 ± 0.391.

No statistically significant difference was observed between these two evaluation settings (Mann-Whitney U test with Bonferroni correction, p-value = 0.4803). This finding indicates that the screening performance of all evaluated approaches in the concatenated setting was comparable to that observed on the individual folds, further supporting the reliability and stability of our evaluation framework.

The concatenated-setting NEF1% of the ligand-derived physicochemical RDKit descriptor-based logistic regression model was 0.070. By comparison, the 16 CBS-specific AI approaches still achieved significantly higher NEF1% values in this setting (one-sample Wilcoxon signed-rank test, p-value = 0.0004), demonstrating that their superior performance relative to simple ligand-based models was preserved in this concatenated analysis and was consistent with the fold-wise results reported in S1.1.
